# Drug–Drug Interactions of 257 Antineoplastic and Supportive Care Agents With 7 Anticoagulants: A Comprehensive Review of Interactions and Mechanisms

**DOI:** 10.1177/1076029620936325

**Published:** 2020-08-31

**Authors:** Érique José F. Peixoto de Miranda, Thamy Takahashi, Felipe Iwamoto, Suzete Yamashiro, Eliana Samano, Ariane Vieira Scarlatelli Macedo, Eduardo Ramacciotti

**Affiliations:** 1 Cardiology, Medical Affairs, Bayer Brasil SA, Sao Paulo, Brazil; 2 Medical Information, Medical Affairs, Bayer Brasil SA, Sao Paulo, Brazil; 3 Cardio-Oncology Outpatient Clinic of Santa Casa de São Paulo, Sao Paulo, Brazil; 4 Vascular Surgery, Hospital e Maternidade Dr. Christovão da Gama, Santo André, Sao Paulo, Brazil; 5 Thrombosis and Haemostasis, Loyola University Medical Center, Chicago, IL, USA.

**Keywords:** drug–drug interactions, DOACs, anticoagulants, antineoplastic agents, supportive care drugs

## Abstract

Data on drug–drug interactions (DDI) of antineoplastic drugs with anticoagulants is scarce. We aim to evaluate factors associated with DDI of antineoplastic and supportive care drugs with anticoagulants resulting in modification of pharmacokinetics of these last mentioned. A literature review on DDI databases and summaries of products characteristics (SmPC) was done. Drug–drug interactions of 257 antineoplastic and supportive care drugs with direct oral anticoagulants (DOACs), warfarin, enoxaparin, or fondaparinux were categorized as no clinically significant expected DDI, potentially weak DDI, potentially clinically significant DDI, and recommendation against coadministration. Logistic regression models were performed to analyze the association between the dependent variable potentially clinically significant interaction/recommendation against coadministration and the mechanisms of DDI. Of the 1799 associations, 84.4% were absence of DDI, 3.6% potentially weak DDI, 10.2% potentially clinically relevant DDI, and 2.0% recommendation against coadministration. Warfarin has higher DDI potential than other anticoagulants. Enoxaparin and fondaparinux have fewer DDI than others. There was no difference between DOACs. Drug–drug interactions with apixaban and rivaroxaban was independently associated with the absence of CYP3A4 competition, P-glycoprotein inhibition, CYP3A4 induction, and drug class of tyrosine kinase inhibitors. Drug–drug interactions with dabigatran and edoxaban was associated with inhibition of P-glycoprotein and tyrosine kinase inhibitors. Warfarin, induction of CYP3A4, and inhibition of CYP2C9. Enoxaparin and fondaparinux, only tyrosine kinase inhibitors. Direct oral anticoagulants did not differ regarding DDI with antineoplastic agents. Warfarin presented more DDI than other anticoagulants. P-glycoprotein inhibition and CYP3A4 induction were independently associated with DDI of antineoplastic agents with DOACs.

## Introduction

Patients with cancer are at increased risk of venous thromboembolism (VTE), recurrence of VTE, and treatment-emergent bleeding in comparison to the general population.^1,2^ Guidelines suggest assessing the potential for pharmacokinetic (PK) drug–drug interaction (DDI) before prescribing anticoagulant to treat cancer-associated thrombosis (CAT).^3,4^ Current data^4^ support the use of low-molecular-weight heparin (LMWH),^5^ rivaroxaban,^6^ and edoxaban^7^ for the treatment of CAT.

Patients with cancer often experience narrow therapeutic index polypharmacy and undergo treatment of several simultaneous comorbidities, such as infectious, respiratory and cardiovascular ones, besides cancer treatment regimens, supportive care, hormonal agents, and targeted cancer therapies.^8–14^ The risk of pharmaceutical, PK, pharmacodynamic (PD), or combined DDI is high. Besides, DDI is associated with adverse events in 20% to 30% of cases, which may be clinically relevant in 80% of patients.^9,10^

Concerns on DDI for CAT management include decreased efficacy (increasing VTE recurrences) and bleeding risk. Drug–drug interaction is categorized into pharmaceutical, PK, PD, or a combination of them.^9,10^ Pharmaceutical interactions result when 2 or more drugs interact because they are incompatible either physically or chemically.^9,10^ Pharmacodynamic DDI occurs when the pharmacological effect of one drug is altered by that of another drug in a combination regimen, such as toxic effects or antitumor activity among anticancer drugs.^9,10^ Pharmacodynamic DDIs are classified into additive, synergistic, antagonistic, or time dependent.

Pharmacokinetic DDI occurs by interfering with the kinetic process of the absorption, distribution, metabolism, and elimination of drugs.^14^ It results from the interference of activity of transporters and biotransformation enzymes in the liver and other extrahepatic tissues, such as the small intestine, and competition for binding at the plasma protein site, when 2 or more drugs are coadministered.

However, despite guidelines of CAT that recommend evaluation of PK DDI, a study of 302 patients with cancer and 603 DDI showed that up to 80% of them are PD.^12^ In the context of patients with cancer, myelotoxicity (particularly thrombocytopenia) and emetogenicity that potentially reduce the absorption of oral anticoagulants are examples of this type of DDI.

Although less frequent than PD,^9,10,12^ PK DDIs are relevant since they may cause large (>5-fold) and abrupt variations in plasma concentrations depending on coadministered therapy. Pharmacokinetic DDIs are clinically significant when single biotransformation pathway is involved; in case of potent inhibition or induction of metabolizing enzyme; variations due to individual genetic polymorphism; abrupt dose–response curve or narrow therapeutic index of medications involved; deviation of metabolic pathway resulted from inhibition or induction that causes accumulation of toxic metabolites; or in case of nonlinear PK.^10^

Data on DDI of antineoplastic and supportive care drugs with anticoagulants are scarce based on the extrapolation of in vivo or in vitro tests with sensitive index substrates.^8–14^ This study aims to review the literature and to assess the consistency of different databases used to evaluate DDI of antineoplastic and supportive care drugs with anticoagulants, helping clinicians to define better strategies for the clinical management of CAT.

## Materials and Methods

### Data Collection

We performed a comprehensive search for DDI of antineoplastic and supportive care agents with anticoagulants (apixaban, dabigatran, enoxaparin, edoxaban, fondaparinux, rivaroxaban, and warfarin) on Drugs.com,^15^ Medscape,^16^ Lexicomp (UptoDate),^17^ and Cancer Drug Interactions (Radboud UMC and University of Liverpool)^18^ to identify the DDI. We also included in our review 264 summaries of product characteristics of antineoplastic and summaries of products characteristics (SmPC), DrugBank,^19^ SuperCYP,^20^ 2018 European Heart Rhythm Association Practical Guide on the use of non-vitamin K antagonist oral anticoagulants in patients with atrial fibrillation (AF),^21^ and review study by Short and Connors to describe the mechanisms of DDI.^22^

Furthermore, we performed a thorough review at Pubmed using key words “anticoagulants” AND “apixaban” AND “dabigatran” AND “edoxaban” AND “rivaroxaban” AND “warfarin” AND “enoxaparin” AND “fondaparinux” AND “clinical trial” OR “case reports” OR “case series” to evaluate further reports of DDI of antineoplastic and supportive care agents with anticoagulants in vivo, without any exclusion criteria.

Interactions were categorized as (1) no clinically significant DDI expected (green); (2) potentially weak DDI (yellow)—additional monitoring action and/or dose adjustment is generally not recommended; (3) potentially clinically significant DDI (orange)—risk–benefit balance, monitoring and/or dose adjustment is generally recommended; and (4) recommendation against coadministration (red) based on a worst-case scenario analysis after consulting databases.^15–18^ Therefore, the color code was based on available databases. This is not the author’s recommendation.

Criteria used to define weak, moderate, or strong inhibition or induction of CYP3A4 (or others) and P-glycoprotein followed the recommendations of the European Medicines Agency^23^ and Food and Drug Administration^24^ guidance for the pharmaceutical industry, considering mechanisms after consulting databases.^19-^ ^22^ Clinical studies (in vivo) using sensitive index substrates for cytochrome P450 pathways or P-glycoprotein were included. Weak, moderate, or strong inhibition corresponds to an area under the curve (AUC_0-infinite_) increase in a given sensitive index substrate up to ≥1.5- to <2-fold, ≥2- to <5-fold, or ≥5-fold, respectively. Strong, moderate, or weak induction were ≥80%, ≥50% to <80% or ≥20% to <50% reduction in AUC, respectively.^23,24^

Furthermore, data on myelotoxicity, frequent myelotoxicity (> 10% of patients), treatment-related thrombocytopenia, and emetogenicity were obtained from the SmPC. Information on hematological toxicity was categorized as “yes” or “no,” while emetogenicity information was categorized according to the frequency of emesis reported by the SmPC: absent, minimum or level 1 (<10%), low or level 2 (10%-30%), moderate or level 3 to 4 (> 30%-90%), and high (>90%).

### Statistical Analysis

As many antineoplastic and supportive care agents as possible were included to allow 80% statistical power, 5% alpha error, and to detect an odds ratio (OR) of 1.55 for the association between clinically relevant DDI (defined as the sum of categories potentially clinically relevant DDI, orange, and recommendation against coadministration, red). The estimated sample was 257 antineoplastic and supportive care agents.

Values were presented as absolute and relative frequency. To assess the difference, Pearson χ^2^ test was used. After collecting information about DDI, data were compiled into spreadsheets. Data on clinically significant DDI (binary variable: potentially clinically significant interaction and recommendation against coadministration = 1 vs absence of expected DDI and potentially weak DDI = 0) of antineoplastic and supportive care agents with each anticoagulant were analyzed one-by-one as dependent variable using unadjusted and multivariate binary logistic regression models. From now on, we will describe the dependent variable as “clinically significant interaction,” as a surrogate of potentially clinically significant interaction and recommendation against coadministration.

For direct oral anticoagulants (DOACs; apixabana, dabigatran, edoxaban, and rivaroxaban), independent variables included were CYP3A4 and P-glycoprotein substrate (both categorized as yes = 1, no = 0); CYP3A4 and P-glycoprotein inhibition (both ordinals categorized as absent = 0, weak = 1, moderate = 2 and strong = 3); and CYP3A4 and P-glycoprotein induction (both ordinals categorized as absent = 0, weak = 1 moderate = 2 and strong = 3), in addition to competition from CYP3A4 and P-glycoprotein (categorized as yes = 1 and no = 0) and tyrosine kinase inhibitors (categorized as yes = 1 and no = 0, or the other oncological drugs classes). All ordinal variables entered the model as numerical variables because of the small number of variables in some strata.

For enoxaparin and fondaparinux, models evaluated the association between clinically significant DDI as the dependent variable and tyrosine kinase inhibitors as the independent variable, adjusted for frequent myelotoxicity. For warfarin, inhibition and induction of CYP3A4, CYP1A2, CYP2C8, CYP2C9, CYP2C19, and tyrosine kinase inhibitors were evaluated.

Values were presented as ORs and 95% CIs. To assess the accuracy of the model, the C-statistics method was used using the model’s predicted probability values, in addition to the Akaike (AIC) and Bayesian (BIC) information criteria. *P* value <.05 was considered statistically significant. All tests were 2-tailed. Analysis were performed using SPSS version 24.0 software for Windows (IBM).

## Results

We included data on 7 comprehensive DDI databases and 264 SmPC (Supplementary Table 1). Furthermore, we did not identify any observational study but 54 case reports and case series that evaluated complex regimens of chemotherapy and anticoagulants. Most of the case reports were related to DDI of antineoplastic and supportive care agents with warfarin (n = 50).

Of the 257 antineoplastic and supportive care drugs, 44 (17.1%) are supportive care, 35 (13.6%) tyrosine kinase inhibitors, 25 (9.7%) monoclonal antibodies, 25 (9.7%) hormonal agents, 21 (8.2%) immune-modulating agents, 19 (7.4%) antimetabolites, 18 (7%) alkylating agents, 11 (4.3%) pyrimidine analogs, 9 (3.5%) anthracyclines/anthracenediones, 7 (2.7%) checkpoint inhibitors, 5 (1.9%) purine analogs, and 5 (1.9%) topoisomerase inhibitors. Of the total, 185 (72%) have some degree of myelotoxicity of which 175 (68.1%) defined as frequent toxicity (>10%) and 149 (58%) with frequent thrombocytopenia. In addition, 76 (29.6%), 91 (35.4%), 42 (16.3%), and 6 (2.3%) drugs have minimal (level 1), low (level 2), moderate (levels 3 and 4), and high emesis potential (level 5), respectively. The full description can be found in Supplementary Table 1.

As substrates of CYP3A4 and P-glycoprotein, 141 (54.9%) and 111 (43.2%) oncological medications were identified, respectively. Of the total, 92 concomitant substrates of CYP3A4 and P-glycoprotein were identified, representing 35.8% of the total or 83% of all substrates of P-glycoprotein and 65% of CYP3A4.

**Figure 1.**
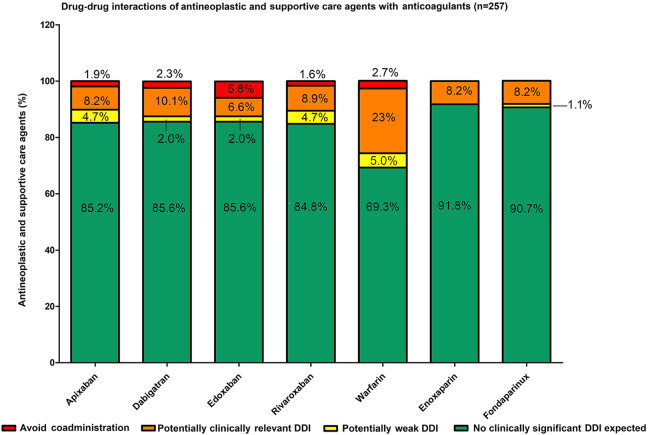
Drug–drug interactions (DDI) between antineoplastic agents and anticoagulants: Most of them are not clinically significant expected (green). Comparison between DOACs resulted: χ^2^ = 0.09; *P* = .993 and between fondaparinux, enoxaparin, and DOACs, χ^2^ = 7.63; *P* = .106. Only comparisons between warfarin and DOACs (χ^2^ = 36.12; *P* < .001) as well as between warfarin, enoxaparin, and fondaparinux (χ^2^ = 45.22; *P* < .001) were statistically significant. DOACs indicates direct oral anticoagulants.

Of the 1799 associations evaluated, there were 84.4% no clinically significant DDI expected, 3.6% of potentially clinically weak DDI, 10.2% of potential clinically relevant DDI, and 2.0% of recommendation against coadministration. We found no clinically significant DDI expected of 219 (85.2%), 220 (85.6%), 220 (85.6%), 218 (84.8%), 178 (69.3%), 236 (91.8%), and 233 (90.7%) antineoplastic and supportive care agents with apixaban, dabigatran, edoxaban, rivaroxaban, warfarin, enoxaparin, and fondaparinux, respectively. Drug–drug interactions that resulted in recommendation against coadministration were 5 (1.9%), 6 (2.3%), 15 (5.8%), 4 (1.6%), 4 (1.6%), 7 (2.7%), 0, and 0, respectively. There was no statistical difference between DOACs or between DOACs and enoxaparin or fondaparinux but only between DOACs and warfarin and between warfarin and enoxaparin and fondaparinux (*P* < .001) regarding interaction with antineoplastic and supportive drugs (Figure 1).

The only drug class most associated with DDI were tyrosine kinase inhibitors: rivaroxaban or apixaban (n = 15 each), dabigatran or edoxaban (n = 17 each), warfarin (n = 19), enoxaparin (n = 9), or fondaparinux (n = 9 each). There were detected clinically significant DDI between all anticoagulants and acalabrutinib, cobimetinib, dasatinib, itrutinib, imatinib, nintedanib, ponatinib, tizovanib, and trametinib.

Unadjusted models of DOACs are presented in Table 1. Adjusted models (Table 2) identified that rivaroxaban and apixaban were independently associated with competition of CYP3A4 (OR = 0.08, 95% CI: 0.01-0.58 and OR = 0.08, 95% CI: 0.01-0.61), CYP3A4 induction (OR = 5.22, 95% CI: 2.17-12.54 and OR = 4.63, 95% CI: 2.03-10.57), P-glycoprotein inhibition (OR = 2.14, 95% CI: 1.17-91.0 and OR = 2.05, 95% CI: 1.17-3.59), and tyrosine kinase inhibitors (OR = 6.60, 95% CI: 2.08-20.95 and OR = 4.63, 95% CI: 2.03-10.57), respectively. Inhibition of CYP3A4 (OR = 2.19, 95% CI: 1.03-4.63) was found as an independent factor for apixaban only. Interaction with edoxaban and dabigatran was independently associated with CYP3A4 competition (OR = 0.09, 95% CI: 0.01-0.99 and OR = 0.09, 95% CI: 0.01-0.99), inhibition of P-glycoprotein (OR = 4.87, 95% CI: 2.17-10.93 and OR = 4.87, 95% CI: 2.17-10.93) and tyrosine kinase inhibitors (OR = 8.67, 95% CI: 2.65-28.38 and OR = 8.67, 95% CI: 2.65-28.38), respectively. There was a trend of association with P-glycoprotein induction (OR = 18.56, 95% CI: 0.85-404.76) and DDI in models of edoxaban and dabigatran. Statistics of goodness of fit are shown (Table 2).

**Table 1.** Unadjusted Logistic Models of Clinically Relevant DDI as the Dependent Variable for Each DOAC.

	Rivaroxaban			Apixaban			Edoxaban		Dabigatran	
	Unadjusted OR (95% CI)	*P* value	Unadjusted OR (95% CI)		*P* value	Unadjusted OR (95% CI)		*P* value	Unadjusted OR (95% CI)	*P* value
CYP3A4 substrate	7.73 (2.26-26.38)	.001	7.34 (2.15-25.13)		.001	9.75 (2.89-32.94)		<.001	9.75 (2.89-32.94)	<.001
P-glycoprotein substrate	2.46 (1.08-5.61)	.032	2.29 (1.00-5.27)		.051	3.36 (1.52-7.44)		.003	3.36 (1.52-7.44)	.003
CYP3A4 competition	3.31 (1.26-8.70)	.015	2.71 (1.00-7.44)		.053	3.24 (1.29-8.11)		.053	3.24 (1.29-8.11)	.012
P-glycoprotein competition	3.07 (0.78-12.11)	.109	0.80 (0.10-6.46)		.834	12.32 (3.64-41.73)		<.001	12.32 (3.64-41.73)	<.001
CYP3A4 inhibition	3.30 (1.91-5.71)	<.001	2.99 (1.74-5.17)		<.001	3.15 (1.87-5.30)		<.001	3.15 (1.87-5.30)	<.001
CYP3A4 induction	2.87 (1.78-4.62)	<.001	2.80 (1.74-4.50)		<.001	1.79 (1.12-2.84)		.014	1.79 (1.12-2.84)	.014
P-glycoprotein inhibition	2.98 (1.94-4.58)	<.001	2.04 (1.34-3.10)		.001	7.28 (3.80-13.96)		<.001	7.28 (3.80-13.96)	<.001
P-glycoprotein induction	1.58 (0.80-3.13)	.191	1.20 (0.52-2.77)		.662	5.76 (1.99-16.69)		.001	5.76 (1.99-16.69)	.001
Tyrosine kinase inhibitors	13.13 (5.41-31.86)	<.001	14.39 (5.83-35.50)		<.0001	13.03 (5.60-30.34)		<.001	13.03 (5.60-30.34)	<.001
Hormonal agents	1.73 (0.55-5.48)	.351	1.24 (0.34-4.46)		.743	0.59 (0.13-2.61)		.483	0.59 (0.13-2.61)	.483

Abbreviations: OR, odds ratio; DDI, drug–drug interactions; DOAC, non-vitamin K antagonist anticoagulants.

**Table 2.** Adjusted Logistic Models of Clinically Relevant DDI as the Dependent Variable for Each DOAC.^a^

	Rivaroxaban		Apixaban		Edoxaban		Dabigatran	
	Adjusted OR (95% CI)	*P* value	Adjusted OR (95% CI)	*P* value	Adjusted OR (95% CI)	*P* value	Adjusted OR (95% CI)	*P* value
CYP3A4 substrate	3.02 (0.59-15.42)	.183	3.56 (0.82-15.44)	.091	3.36 (0.68-16.64)	.138	3.36 (0.68-16.64)	.138
P-glycoprotein substrate	0.82 (0.26-2.60)	.729	1.17 (0.42-3.26)	.771	0.69 (0.20-2.33)	.544	0.69 (0.20-2.33)	.544
CYP3A4 competition	0.08 (0.01-0.58)	.013	0.08 (0.01-0.61)	.015	0.09 (0.01-0.99)	.049	0.09 (0.01-0.99)	.049
P-glycoprotein competition	5.02 (0.19-133.75)	.335	0.01 (0.0-962.8)	.348	0.13 (0.01-8.38)	.337	0.13 (0.01-8.38)	.337
CYP3A4 inhibition	1.84 (0.75-4.52)	.186	2.19 (1.03-4.63)	.041	1.56 (0.63-3.84)	.334	1.56 (0.63-3.84)	.334
CYP3A4 induction	5.22 (2.17-12.54)	<.001	4.63 (2.03-10.57)	<.001	1.37 (0.52-3.65)	.528	1.37 (0.52-3.65)	.528
P-glycoprotein inhibition	2.14 (1.17-3.91)	.014	2.05 (1.17-3.59)	.013	4.87 (2.17-10.93)	<.001	4.87 (2.17-10.93)	<.001
P-glycoprotein induction	0.46 (0.09-2.31)	.346	4.95 (0.06-426.65)	.482	18.56 (0.85-404.76)	.063	18.56 (0.85-404.76)	.063
Tyrosine kinase inhibitors	6.60 (2.08-20.95)	.001	4.63 (2.03-10.57)	<.001	8.67 (2.65-28.38)	<.001	8.67 (2.65-28.38)	<.001

Abbreviations: AIC, Akaike; BIC, Bayesian; DDI, drug–drug interactions; DOAC, non-vitamin K antagonist anticoagulants; OR, odds ratio.

^a^ Models: rivaroxaban: BIC = 139.7, AIC = 104.2, C-statistics 0.91 (95% CI: 0.85-0.97), *P* < .001; apixaban: BIC =138.4, AIC = 102.9, C-statistics 0.86 (95% CI: 0.78-0.93), *P* < .001; edoxaban: BIC = 125.41, AIC = 89.9, C-statistics 0.93 (95% CI: 0.88-0.98), *P* < .001; dabigatran: BIC = 125.41, AIC = 89.9, C-statistics 0.93 (95% CI: 0.88-0.98), *P* < .001.

Enoxaparin and fondaparinux were independently associated with drug class of tyrosine kinase inhibitors (OR = 5.88, 95% CI 2.24-15.40), adjusted for frequent myelotoxicity (Table 3); warfarin with CYP2C9 inhibition (OR = 6.89, 95% CI: 1.93-24.61) and CYP3A4 induction (OR = 2.50, 95% CI: 1.24-5.03; Table 4). Tyrosine kinase inhibitors are independently associated with inhibition of P-glycoprotein (OR = 3.99, 95% CI: 2.16-7.38) and CYP3A4 substrate (OR = 5.20, 95% CI: 1.29-20, 89; Supplementary Table 2).

**Table 3.** Unadjusted and Adjusted Logistic Models of Clinically Relevant DDI as the Dependent Variable for Enoxaparin and Fondaparinux.^a^

	Unadjusted model		Adjusted model	
	OR (95% CI)	*P* value	OR (95% CI)	*P* value
Tyrosine kinase inhibitors	6.06 (2.33-15.75)	<.001	5.88 (2.24-15.40)	<.001
Frequent myelotoxicity	1.55 (0.55-4.39)	.409	1.28 (0.44-3.75)	.654

Abbreviations: OR, odds ratio, DDI, drug–drug interaction.

^a^ Adjusted models’s C-statistics: 0.65 (95% CI: 0.50-0.79), *P* = .028.

**Table 4.** Unadjusted and Adjusted Logistic Models of Clinically Relevant DDI as the Dependent Variable for Warfarin.^a^

	Unadjusted model		Adjusted model	
	OR (95% CI)	*P* value	OR (95% CI)	*P* value
CYP3A4 inhibition	3.26 (2.05-5.19)	<.001	1.56 (0.85-2.88)	.156
CYP3A4 induction	3.88 (2.27-6.64)	<.001	2.50 (1.24-5.03)	.010
CYP1A2 inhibition	1.98 (0.94-4.18)	.074	1.12 (0.46-2.74)	.805
CYP2C8 inhibition	3.68 (1.93-6.99)	<.001	1.07 (0.43-2.68)	.890
CYP2C9 inhibition	7.61 (3.39-17.07)	<.001	6.89 (1.93-24.61)	.003
CYP2C19 inhibition	4.96 (2.01-12.24)	.001	0.48 (0.11-2.12)	.333
CYP1A2 induction	3.08 (0.96-9.92)	.059	0.84 (0.17-4.16)	.834
CYP2C8 induction	22.54 (2.72-186.97)	.004	5.55 (0.49-62.68)	.166
CYP2C9 induction	16.61 (1.99-138.65)	.009	1.38 (0.12-15.73)	.796
CYP2C19 induction	6.83 (1.36-34.15)	.019	2.05 (0.23-18.67)	.523
Tyrosine kinase inhibitors	4.42 (2.11-9.26)	<.001	2.53 (0.97-6.59)	.057
CYP3A4 substrate	4.26 (2.21- 8.20)	<.001	-	-
CYP1A2 substrate	2.52 (1.20-5.27)	.014	-	-
CYP2C8 substrate	4.39 (1.93- 9.97)	<.001	-	-
CYP2C9 substrate	3.45 (1.58-7.53)	.002	-	-
CYP2C19 substrate	4.04 (1.59-10.26)	.003	-	-
CYP3A4 competition	6.14 (2.72-13.86)	<.001	-	-
CYP1A2 competition	2.92 (0.18-47.40)	.450	-	-
CYP2C8 competition	5.94 (0.53-66.58)	.419	-	-
CYP2C9 competition	2.92 (0.18-47.40)	.450	-	-
CYP2C19 competition	2.92 (0.18-47.40)	.450	-	-

Abbreviations: OR, odds ratio; DDI, drug–drug interaction.

^a^ Adjusted models’s C-statistics: 0.83 (95% CI: = 0.77-0.90), *P* < .001.

## Discussion

The use of DOACs has been increasing, including in patients with cancer, for VTE management or stroke prevention in AF, and potential DDI is a major concern. This present study provides complementary information to the European Heart Rhythm Association’s 2018 recommendations on the use of DOACs in patients with AF^21^ and the review by Short and Connors.^22^ Warfarin has a higher DDI potential than DOACs, a LMWH, and a pentasaccharide (fondaparinux) anticoagulant. Few among nearly 1800 associations have a recommendation for non-coadministration.

Due to the large promiscuity of interaction with cytochrome P450 isoenzymes, the chance of DDI of antineoplastic and supportive care drugs with warfarin compared to other anticoagulants was higher.^25^ In addition, case reports or case series of fluctuations in international normalized ratio (INR) values and possible DDI have been described between warfarin and several anticancer drugs (Supplementary Table 1). Clinical reports of possible DDI between antineoplastic and supportive care drugs and other anticoagulants are rare (Supplementary Table 1).

Statistical models had high internal validity and were also consistent with the literature. Factors associated with DDI between antineoplastic and supportive care drugs and rivaroxaban and apixaban were the absence of CYP3A4 competition, CYP3A4 induction, P-glycoprotein inhibition, and CYP3A4 inhibition (for apixaban only). There was a trend of association between CYP3A4 inhibition and rivaroxaban. The fraction metabolized by CYP3A4 fraction (fmCYP_3A4_) of apixaban (25%)^26^ is slightly higher than rivaroxaban (18%).^27^

Factors associated with DDI of antineoplastic and supportive care drugs with dabigatran and edoxaban were the absence of CYP3A4 competition, CYP3A4 induction, and P-glycoprotein inhibition. There was a trend of association with P-glycoprotein induction. CYP3A4 and P-glycoprotein substrates and CYP3A4 and P-glycoprotein competition were important confounders in this analysis. After adjustment, there was a Simpson paradox or inversion of the association for CYP3A4 competition and loss of significance of substrate of CYP and P-glycoprotein.

This analysis also showed that tyrosine kinase inhibitors have higher potential for DDI with all anticoagulants evaluated, except for warfarin, in comparison to other classes of antineoplastic and supportive care drugs. The strength of association was higher for DOACs, drugs that are a substrate for P-glycoprotein and CYP3A4. Tyrosine kinase inhibitors are mostly substrate and/or inhibitors of CYP3A4 and P-glycoprotein, which ensures a relatively high oral bioavailability.^11,13^ Despite the lower association strength of PK DDI of antineoplastic and supportive care drugs with warfarin, high protein binding rate of warfarin and tyrosine kinase inhibitors, both> 90%, which results in competition, has not been taken into account in this model.^11,13,28^ Also, tyrosine kinase inhibitors are associated with an increased risk of bleeding and thrombocytopenia and may interact with enoxaparin and fondaparinux.

Drug–drug interaction of anticoagulants with antineoplastic and supportive care drugs resulting in increasing or decreasing levels of cancer drugs is not clinically significant, since DOACs, fondaparinux, and enoxaparin do not induce or inhibit cytochrome P450 or P-glycoprotein. Furthermore, neither anticoagulants are associated with QTc^29–32^ interval prolongation, nor does it potentiate the effect of drugs that cause it, such as some tyrosine kinase inhibitors. Of great concern to oncologists and their patients are the DDI of anticoagulants with antineoplastic and supportive care drugs that result in modification of the effect of anticancer drugs, particularly tyrosine kinase inhibitors, due to serious adverse events.

All DOACs, as shown in this study, have a similar DDI profile with antineoplastic and supportive care drugs. It must be explained by the low fraction metabolization of CYP3A4 DOACs and the wide spectrum overlapping of CYP3A4 and P-glycoprotein substrates. A large number of in vitro and in vivo studies^33–39^ have established a synergism between phase I metabolism by intestinal CYP3A4/5 and P-glycoprotein, present at high levels in enterocyte villi in the gastrointestinal tract, whose activity results in the active extrusion of absorbed drug.^36^ Therefore, this activity is a major determinant of the systemic bioavailability of orally administered drugs. This synergism between CYP3A4 and P-glycoprotein is an adaptive response that makes the gastrointestinal tract a barrier to xenobiotic absorption.^34,35^ In contrast, modulation of this mechanism may improve the bioavailability of oral drugs.

Although the difference is not statistically significant, LMWH and a pentasaccharide anticoagulant had less clinically significant DDI with antineoplastic and supportive care drugs than DOACs^3,4,40^: Both do not undergo cytochrome P450 metabolism and are not P-glycoprotein substrates. Therefore, the recommendation of CAT guidelines^3,4^ that LMWH is the preferred therapy in cases of clinically significant DDI of anticancer therapies and supportive drugs with anticoagulants is consistent with the findings of this study.

However, PD DDI, which is much more frequent in patients with cancer, should be considered in addition to PK.^12^ Modern targeted cancer therapies such as ipilimumab and various tyrosine kinase inhibitors are associated with increased risk of bleeding and interaction with all anticoagulants regardless of interaction with cytochrome P450 or P-glycoprotein. Also, most cancer medications are associated with myelotoxicity, including high rates of thrombocytopenia, and the high frequency of vomiting, which interferes with the adherence and/or absorption of oral therapies. Therefore, monitoring of platelets count, liver function, renal function, factor Xa, and serum anticoagulant levels is necessary for situations in which an anticoagulant cannot be replaced.

This study has limitations. Although the model of factors associated with DDIs from mechanisms detected in vivo with sensitive indexes substrates for each pathway is highly accurate, there are few clinical studies to date that have evaluated the PK of anticoagulants in patients with cancer undergoing combined drug treatment. This is not a systematic review. Nevertheless, we included as much as possible references on comprehensive DDI databases and SmPC based on worst-case scenario.

To date, there is no subanalysis of pivotal clinical studies of CAT treatment evaluating DDI of antineoplastic and supportive care drugs regimens with anticoagulants. In HOKUSAI-VTE-Cancer,^7^ 25% of patients were eligible for a low dose of edoxaban, whose criteria included, out of 2 others, coadministration of P-glycoprotein inhibitory drugs. However, patients using high interaction potential cancer therapies, such as tyrosine kinase inhibitors (18 or 3.4% of patients in each study arm), were not evaluated for a stand-alone DDI outcome. This study also did not evaluate the synergistic effect on a potential DDI resulting from the combination of several drugs in cancer regimens.

To date, few clinical studies have evaluated the safety of anticoagulants in patients with cancer in the context of multiple combined cancer therapies. A pilot clinical study (NCT02921022)^41^ evaluated the safety of rivaroxaban in 42 patients with advanced pancreatic adenocarcinoma and Karnofsky performance status ≥70% using gemcitabine, nab-paclitaxel, and PEGPH20 (PAG). The rate of thromboembolic events was 1 (2.4%) and major bleeding 2 (4.8%) at a follow-up of 10.9 months.

However, ongoing studies aim to address some of these limitations. COSIMO registry will include DDI as a secondary outcome.^42^ A prospective, observational, and hypothesis-generating international registry from the Internal Society of Thrombosis and Hemostasis (ISTH)^43^ was designed to assess the safety and efficacy of 200 patients with cancer followed for 6 months on concomitant use of DOACs and antineoplastic and supportive care drugs. Targeted therapies include ibrutinib, acalabrutinib, imatinib, nilotinib, osimertinib, alectinib, sunitinib, cabozantinib, lapatinib, palbocyclib, vemurafenib, or everolimus. The 200 patients, 100 of whom will be diagnosed with VTE and 100 AF, will be descriptively analyzed for recurrent VTE events, ischemic stroke, bleedings (major or clinically relevant non-major), cancer status, and all-cause mortality. Despite limitations, this study brings important information supporting physicians in clinical practice and providing the best patient care.

In conclusion, antineoplastic and supportive care drugs have fewer clinically significant DDI with DOACs, enoxaparin, and fondaparinux than with warfarin. Although the number of interactions of antineoplastic and supportive care drugs with enoxaparin and fondaparinux was lower than DOACs, the difference was not statistically significant. Low-molecular-weight heparin is the preferred therapy in cases of clinically significant DDI of antineoplastic and supportive care drugs with anticoagulants, according to guidelines.^3,4^

Furthermore, there were no differences in DDI of antineoplastic and supportive care drugs with DOACs, despite the lower potential of PK DDI of edoxaban with CYP3A4 substrates, likewise, dabigatran that does not interact with it. This is explained by the large overlapping of CYP3A4 and P-glycoprotein substrates spectra. Furthermore, apixaban and rivaroxaban have a similar fraction metabolized by CYP3A4. All DOACs, as shown in this study, have a similar DDI profile with antineoplastic and supportive care drugs, especially regarding interaction with tyrosine kinase inhibitors. P-glycoprotein inhibition and CYP3A4 induction were independently associated with DDI of antineoplastic and supportive care agents and DOACs.

## Supplemental Material

Supplemental Material, DDI_between_anticoagulants_and_antineoplastic_Appendix - Drug–Drug Interactions of 257 Antineoplastic and Supportive Care Agents With 7 Anticoagulants: A Comprehensive Review of Interactions and Mechanisms Click here for additional data file. Supplemental Material, DDI_between_anticoagulants_and_antineoplastic_Appendix for Drug–Drug Interactions of 257 Antineoplastic and Supportive Care Agents With 7 Anticoagulants: A Comprehensive Review of Interactions and Mechanisms by Érique José F. Peixoto de Miranda, Thamy Takahashi, Felipe Iwamoto, Suzete Yamashiro, Eliana Samano, Ariane Vieira Scarlatelli Macedo and Eduardo Ramacciotti in Clinical and Applied Thrombosis/Hemostasis
