# Supplementary material for: Drug–Drug Interactions of 257 Antineoplastic and Supportive Care Agents With 7 Anticoagulants: A Comprehensive Review of Interactions and Mechanisms
Source: Clin Appl Thromb Hemost. 2020 Aug 31;26:1076029620936325. doi: 10.1177/1076029620936325 (PMC7466894; doi:10.1177/1076029620936325)
Supplement: Supplemental Material, DDI_between_anticoagulants_and_antineoplastic_Appendix - Drug–Drug Interactions of 257 Antineoplastic and Supportive Care Agents With 7 Anticoagulants: A Comprehensive Review of Interactions and Mechanisms [file DDI_between_anticoagulants_and_antineoplastic_Appendix.pdf]

Appendix

Supplementary Table 1. Drug-Drug Interactions[265-273] of Antineoplastic and Supportive Care Agents[1-257] with Anticoagulants[258-264]

Antineoplastic Agent	Drug Class	CYP3A4	CYP1A2	CYP2C8	CYP2C9	CYP2C19	P-glycoprotein	Myelotoxicity	Emetogenicity**	Apixaban[258]	Dabigatran[259]	Edoxaban[260]	Rivaroxaban[261]	Warfarin[262]	Enoxaparin[263]	Fondaparinux[264]
5-fluorouracil[1]	Pyrimidine analogs		S ↓	S	S↓			Yes	2							
Abemaciclib[2]	CDK 4 and 6 Inhibitors	S					↓	Yes	2							
Abiraterone[3]	Hormonal agents	S ↓	↓↓↓	↓	↓	↓	↓↓↓	No	2							
Acalabrutinib[4]	Tyrosine kinase inhibitors	S↑↑/↓		↓↓	↓↓	↓	↓	Yes	2						*	*
Acetate, Megestrol [5]	Hormonal agents	S					↓↓	Yes	1							
Acid, Folinic [6]	Treatment adjuvant							No	1							
Acid, Ibandronic[7]	Supportive care						S	No	No							
Acid, Zoledronic[8]	Supportive care							Yes	2							
Afatinib[9]	Tyrosine kinase inhibitors						S ↓↓↓	No	2							
Alectinib[10]	Tyrosine kinase inhibitors	S ↓/↑		↓			↓	Yes	2							
Alemtuzumab[11]	Monoclonal antibodies							Yes	2							

Allopurinol[12]	Supportive care							No	No							
Altretamine[13]	Alkylanting agents		S↓					Yes	3 a 4							
Amifostine[14]	Supportive care							No	3 a 4							
Aminoglutethimide[15]	Hormonal agents	S	S			S	S	No	2							
Anastrozole[16]	Hormonal agents	↓	↓		↓			Yes	2							
Ansacrine[17]	Topoisomerase inhibitors						↓	Yes	2							
Aprepitant[18]	Supportive care	S↓↓/↑				↑/↓		No	No							
Atezolizumab[19]	Checkpoint inhibitors							No	2							
Avelumab[20]	Checkpoint inhibitors							Yes	2							
Axitinib[21]	Tyrosine kinase inhibitors	S	S			S	S	Yes	2							
Azacitidine[22]	Pyrimidine analogs							Yes	3 a 4							
Azathioprine[23]	Pyrimidine analogs		S					Yes	1					*	*	*
Belinostat[24]	HDAC inhibitors			↓	↓			Yes	1							
Bendamustine[25]	Alkylanting agents	S					S	Yes	3 a 4							
Bevacizumab[26]	Monoclonal antibodies							Yes	1					*		
Bexarotene[27]	Retinoids	S		↓↓	S	↓	S	Yes	2							
Bicalutamide[28]	Hormonal agents	S↓↓			↓	↓		Yes	2							
Bleomycin[29]	Intercalating agents	S					S	No	1							
Blinatumomab[30]	Monoclonal antibodies							Yes	2							

Bortezomib[31]	Proteasome inhibitors	S↓	S↓		S↓	S↓	S	Yes	1							
Bosutinib[32]	Tyrosine kinase inhibitors	S↓	↓				S↓	Yes	2							
Brentuximab vedotin[33]	Monoclonal antibodies	S					S	Yes	1							
Busereline[34]	Hormonal agents							No	1							
Busulfan[35]	Alkylanting agents	S						Yes	3 a 4							
Cabazitaxel[36]	Antimitotic agents	S		S			S↓	Yes	2							
Cabozantinib[37]	Tyrosine kinase inhibitors	S		S↓	S		↓	Yes	2							
Calaspargase pegol[38]	Cytotoxic enzyme							No	1							
Capecitabine[39]	Pyrimidine analogs				S↓			Yes	2							
Carboplatin[40]	Platin-based agents							Yes	3 a 4							
Carfilzomib[41]	Proteasome inhibitors						S	Yes	1							
Carmustine[42]	Alkylanting agents		S					Yes	5							
Cemiplimab[43]	Checkpoint inhibitors							Yes	1							
Ceritinib[44]	Tyrosine kinase inhibitors	S↓			S↓↓		S	Yes	1							
Cetuximab[45]	Monoclonal antibodies							Yes	1							
Cyclophosphamide[46]	Alkylanting agents	S↓↓/		S	S	S		Yes	3 a 4					*	*	*
Cyclosporine[47]	Immune-modulating agents	S↓↓/↑↑		↓	↓	↓	S↓↓↓/↑	Yes	1							
Cyproterone[48]	Hormonal agents	S↓		↓	↓	↓		Yes	No							
Cisplatin[49]	Platin-based agents				↓		S	Yes	5							

Citarabine[50]	Pyrimidine analogs	S						Yes	3 a 4							
Cladribine[51]	Purine analogs							Yes	1							
Clodronate[52]	Supportive care							No	1							
Clofarabine[53]	Purine analogs							Yes	3 a 4							
Clonazepan[54]	Supportive care	S						No	1							
Chlorambucil[55]	Alkylanting agents							Yes	2							
Cobimetinib[56]	Tyrosine kinase inhibitors	S					S	Yes	1						*	*
Codeine[57]	Supportive care	S						No	No							
Crizotinib[58]	Tyrosine kinase inhibitors	S ↓↓					S ↓↓	Yes	3 a 4							
Dabrafenib[59]	Tyrosine kinase inhibitors	S		S ↑/ ↓↓↓			S	Yes	2							
Dacomitinib[60]	Tyrosine kinase inhibitors	S			S		S ↓	Yes	1							
Dacarbazine[61]	Alkylanting agents		S ↓					Yes	5							
Dactinomycin[62]	Intercalating agents						S ↓	Yes	3 a 4							
Daratumumab[63]	Monoclonal antibodies							Yes	2							
Darbepoetin alpha[64]	Supportive care							No	No							
Darolutamide[65]	Hormonal agents	S	S	S	S		S	No	1							
Dasatinib[66]	Tyrosine kinase inhibitors	S ↓	S					Yes	2	*	*	*	*	*	*	*
Daunorubicin[67]	Anthracyclin/Anthracenediones	S ↓/↑	S				S ↓/↑	Yes	3 a 4							
Daunorubicin, liposomal pegylated[68]	Anthracyclin/Anthracenediones	S ↓/↑	S				S ↓/↑	Yes	3 a 4							

Decitabine[69]	Pyrimidine analogs						S	Yes	1							
Denileukin diftitox[70]	Immune-modulating agents							Yes	1							
Denosumab[71]	Monoclonal antibodies							No	1							
Degarelix[72]	Hormonal agents							No	1							
Dexamethasone[73]	Immune-modulating agents	S↑↑↑ /↓	↑	↑	↑	↑	S↑↑↑ /↓	No	No						*	*
Dexrazoxane[74]	Supportive care	S					S↓	Yes	1							
Diazepan[75]	Supportive care	S↓	S↓	S	S↓	S↓	S	No	No							
Diphenhydramine[76]	Supportive care			S	S		S	Yes	No							
Dimenhydrinate[77]	Supportive care	S	↓				S	No	No					*		
Dinutuximab[78]	Monoclonal antibodies							Yes	1							
Dipyrone[79]	Supportive care	S↑						No	No					*		
Docetaxel[80]	Antimitotic agents	S↓					S	Yes	1							
Dolasetron[81]	Supportive care	S			S			No	No							
Doxorubicin[82]	Anthracyclin/Anthracenediones	S↑↑ /↓					S ↑↑↑/↓↓↓	Yes	5							
Doxorubicin, liposomal pegylated[83]	Anthracyclin/Anthracenediones	S ↑↑/↓					S↑↑↑ /↓↓↓	Yes	1							
Dronabinol[84]	Supportive care	S			S	S	↓	No	No					*		
Durvalumab[85]	Checkpoint inhibitors							No	1							
Enzalutamide[86]	Hormonal agents	S↑↑↑ /↓			S ↑↑	↑↑	↓	No	1							
Epirubicin[87]	Anthracyclin/Anthracenediones						S	Yes	3 a 4							

Epoetin alpha[88]	Supportive care							No	No							
Elotuzumab[89]	Monoclonal antibodies							Yes	2							
Erdafitinib[90]	Tyrosine kinase inhibitors	S ↑/↓			S		S ↓	Yes	2							
Eribulin[91]	Antimitotic agents	S					S	Yes	2							
Erlotinib[92]	Tyrosine kinase inhibitors	S↓		S ↓↓			S	No	2					*		
Estreptozocin[93]	Alkylanting agents		↑				↑	Yes	5							
Etoposide[94]	Topoisomerase inhibitors	S ↓/	S	↓	↓		S ↓	Yes	3 a 4							
Estramustine[95]	Alkylanting agents	S ↓				↓	↓	Yes	3 a 4							
Everolimus[96]	Immune-modulating agents	S					S	Yes	2							
Exemestane[97]	Hormonal agents	S						No	1							
Fentanyl[98]	Supportive care	S ↓					↓	No	1							
Filgrastim[99]	Supportive care							No	1							
Floxuridine[100]	Pyrimidine analogs							Yes	1							
Fludarabine[101]	Purine analogs							Yes	2							
Flutamide[102]	Hormonal agents	S	S ↓			S		Yes	1							
Fosaprepitant[103]	Supportive care	S ↓↓/						No	No							
Fotemustine[104]	Alkylanting agents							Yes	2							
Fulvestrant[105]	Hormonal agents	S						Yes	1							

Gefitinib[106]	Tyrosine kinase inhibitors	S↓			↓	↓	S↓	No	2							
Gemcitabine[107]	Pyrimidine analogs						S	Yes	2					*	*	*
Gentuzumab ozogamicin[108]	Monoclonal antibodies							Yes	1							
Gilteritinib[109]	Tyrosine kinase inhibitors	S↓					S	No	2							
Glasdegib[110]	Hedgehog pathway inhibitors	S		↓			S↓	Yes	2							
Granisetron[111]	Supportive care	S					S	No	No							
Goserelin[112]	Hormonal agents							No	1							
Hydrocortisone[113]	Immune-modulating agents	S↑/↓					S	No	No						*	*
Hydromorphone[114]	Supportive care	S		S			S	No	No							
Hydroxyurea[115]	Antimetabolites							Yes	2							
Ibritumomab[116]	Monoclonal antibodies							Yes	2							
Ibrutinib[117]	Tyrosine kinase inhibitors	S↓		↓	↓	↓	↓↓↓	Yes	2						*	*
Idarubicin[118]	Anthracyclin/Anthracenediones	S↓					S	Yes	3 a 4							
Idelalisib[119]	Tyrosine kinase inhibitors	↓↓↓/↑↑↑		↓		↓	S↓	Yes	1							
Ifosfamide[120]	Alkylanting agents	S↓/↑		S	S	S		Yes	3 a 4							
Imatinib[121]	Tyrosine kinase inhibitors	S↓↓	S	S↓	S↓	S	S↓↓	Yes	2						*	*
Inotuzumab ozogamicin[122]	Monoclonal antibodies						S	Yes	2							
Interferon-alpha-2a[123]	Immune-modulating agents							Yes	3 a 4							
Interferon alpha-2b[124]	Immune-modulating agents							Yes	3 a 4							

Interferon alfa-2a pegylated[125]	Immune-modulating agents							Yes	2							
Interferon alpha-2b pegylated[126]	Immune-modulating agents		↓					Yes	2							
Interleukin-2 [127]	Immune-modulating agents	↓						Yes	3 a 4							
Ipilimumab[128]	Checkpoint inhibitors							Yes	1	*	*	*	*	*	*	*
Irinotecan[129]	Topoisomerase inhibitors	S					S	Yes	3 a 4							
Irinotecan, liposomal [130]	Topoisomerase inhibitors	S					S	Yes	3 a 4							
Ixabepilone[131]	Antimitotic agents	S↓						Yes	2							
Ixazomib[132]	Proteasome inhibitors	S	S	S	S	S	S	Yes	2							
Lapatinib[133]	Tyrosine kinase inhibitors	S↓		S↓		S	↓↓↓	Yes	2							
L-Asparaginase[134]	Cytotoxic enzyme							No	1							
Larotrectinib[135]	Agnostic agent	S↓					S	Yes	2							
Lanreotide[136]	Hormonal agents	S↓						No	2							
Lenalidomide[137]	Immune-modulating agents						S	Yes	2							*
Lenvatinib[138]	Tyrosine kinase inhibitors	S↓/↑	↓	↓↓	↓	↓	S	Yes	3 a 4							
Letrozole[139]	Hormonal agents	S						No	1							
Leuprorelin[140]	Hormonal agents	S						Yes	1							
Lenograstim[141]	Supportive care							No	1							
Levoleucovorin[142]	Treatment adjuvant							No	1							
Lomustine[143]	Alkylanting agents	↓						Yes	4							

Lorazepan[144]	Supportive care	S						No	No							
Lutetium 177-dotatate[145]	Hormonal agents							Yes	1							
Mechlorethamine[146]	Alkylanting agents							Yes	5							
Melphalan[147]	Alkylanting agents							Yes	3 a 4							
Mercaptopurine[148]	Pyrimidine analogs							Yes	2							
Mesna[149]	Supportive care							Yes	No							
Methadone[150]	Supportive care	S ↑/↓	S	S	S	S	↓	No	No							
Methylprednisolone[151]	Immune-modulating agents	S					S	No	No						*	*
Metoclopramide[152]	Supportive care		↓					Yes	No							
Methotrexate[153]	Antimetabolites	S					S	Yes	3 a 4							
Midazolam[154]	Supportive care	S ↓						No	No							
Midostaurin[155]	Tyrosine kinase inhibitors	S ↑↑↑/↓↓↓	↑/↓	↑/↓	↑/↓	↑/↓	↓	Yes	2							
Mifamurtide[156]	Immune-modulating agents							Yes	3 a 4							
Mitomycin C[157]	Intercalating agents	S	↑				S	Yes	2							
Mitotane[158]	Hormonal agents	S ↑↑↑	↓		↓	↓		Yes	3 a 4							
Mitoxantrone[159]	Anthracyclin/Anthracenediones	↓					S ↓/↑	Yes	2							
Morphine[160]	Supportive care	S		S			S	No	No							
Nabilone[161]	Supportive care	S ↓		↓↓	↓		S	No	No							
Nab-paclitaxel[162]	Antimitotic agents	S ↓/↑↑		S	S		S ↓	Yes	2							

Nelarabine[163]	Antimetabolites							Yes	1							
Neratinib[164]	Tyrosine kinase inhibitors	S					↓	Yes	2							
Nilotinib[165]	Tyrosine kinase inhibitors	S ↓↓		↓/↑	↓/↑		S ↓↓↓	Yes	2							
Nilutamide[166]	Hormonal agents			↓	↓	↓		Yes	1							
Nimotuzumab[167]	Monoclonal antibodies							Yes	1							
Nintedanib[168]	Tyrosine kinase inhibitors	S					S ↓	Yes	2						*	*
Niraparib[169]	PARP inhibitors	S	S				S ↓	Yes	2						*	*
Nivolumab[170]	Checkpoint inhibitors							Yes	2							
Obinutuzumab[171]	Monoclonal antibodies							Yes	2							
Octreotide[172]	Hormonal agents	S ↓					S	No	2							
Ofatumumab[173]	Monoclonal antibodies							Yes	1							
Olaparib[174]	PARP inhibitors	S ↓					S ↓	Yes	2							
Olaratumab[175]	Monoclonal antibodies							Yes	3 a 4							
Omeprazole[176]	Supportive care	S ↓/↑	↓ /↑	S	S	S	S ↓	No	No							
Ondansetron[177]	Supportive care	S ↓	S ↓		S		S	No	No							
Osimesertinib[178]	Tyrosine kinase inhibitors	S ↓/↑					S ↓	Yes	1							
Oxaliplatin[179]	Platin-based agents		S					Yes	3 a 4							
Oxicodone[180]	Supportive care	S						No	No							
Paclitaxel[181]	Antimitotic agents	S ↓/↑↑		S	S ↓	↓	S ↓	Yes	2							

Palbociclib[182]	CDK 4 and 6 Inhibitors	S↓					S↓	Yes	2							
Palonosetron[183]	Supportive care	S	S					No	No							
Palonosetron e netupitant[184]	Supportive care	S↓↓	S		S		S	No	No							
Pamidronate[185]	Supportive care							No	No							
Panitumumab[186]	Monoclonal antibodies							Yes	1							
Panobinostat[187]	HDAC inhibitors	S↓↓				S	S	Yes	2	*	*	*	*	*	*	*
Paracetamol (Acetaminophen)[188]	Supportive care	S↓/↑	S	S	S		S↓	No	No					*		
Pazopanib[189]	Tyrosine kinase inhibitors	S↓	S	S↓			S	Yes	2							
Pembrolizumab[190]	Checkpoint inhibitors							Yes	2							
Pemetrexed[191]	Antimetabolites							Yes	2							
Pentostatin[192]	Purine analogs	S					S	Yes	1							
Pertuzumab[193]	Monoclonal antibodies							Yes	2							
Pixantrone[194]	Anthracyclin/Anthracenediones		S↓	S↓			S	Yes	2							
Pomalidomide[195]	Immune-modulating agents	S	S				S	Yes	2							*
Ponatinib[196]	Tyrosine kinase inhibitors	S		S↓			S↓↓↓	Yes	2	*	*	*	*	*	*	*
Pralatrexate[197]	Antimetabolites						S	Yes	1							
Prednisone[198]	Immune-modulating agents	S↑↑/↓					S	No	No						*	*
Prenisolone[199]	Immune-modulating agents	S↑↑					S	No	No						*	*
Procarbazine[200]	Alkylanting agents		S					Yes	3 a 4							

Prochlorperazine[201]	Supportive care	S						No	No							
Radium[202]	Radiocompounds							Yes	1							
Raloxifene[203]	Hormonal agents	S↑		↑			S	No	2							
Raltitrexed[204]	Antimetabolites	S					S	Yes	3 a 4							
Ramucirumab[205]	Monoclonal antibodies		S					Yes	2							
Ranitidine[206]	Supportive care	↓	S↓			S	S↓	Yes	No							
Regorafenib[207]	Tyrosine kinase inhibitors	S↓		↓	↓	↓	S↓	Yes	2							
Ribociclib[208]	CDK 4 and 6 Inhibitors	S↓↓	↓				S↓	Yes	2							
Rituximab[209]	Monoclonal antibodies							Yes	2							
Rolapitant[210]	Supportive care	S					↓	No	No					*		
Romidepsin[211]	HDAC inhibitors	S				S	S	Yes	3 a 4					*		
Rucaparib[212]	PARP inhibitors	S↓	S	↓	↓	↓	S↓	Yes	2							
Ruxolitinib[213]	Tyrosine kinase inhibitors	S			S		S↓	Yes	1							
Saccharate, Iron [214]	Supportive care							No	No							
Sargramostim[215]	Supportive care							No	No							
Sirolimus[216]	Immune-modulating agents	S↓					S↓	Yes	1							
Sorafenib[217]	Tyrosine kinase inhibitors	S↓	S	S↓↓↓	↓↓↓	↓	S↓	Yes	2							
Sunitinib[218]	Tyrosine kinase inhibitors	S↓					S↓↓↓	Yes	2					*		
Tacrolimus[219]	Immune-modulating agents	S↓					S↑ /↓↓↓	Yes	2							

Talazoparib[220]	PARP inhibitors							Yes	2							
Thalidomide[221]	Immune-modulating agents			S	S	S↓	S	Yes	2							*
Tamoxifen[222]	Hormonal agents	S↑/↓↓	S	↓↓	S↓	S	S/↓↓↓	Yes	1					*		
Tegafur/gimeracil/oteracil (S1)[223]	Pyrimidine analogs		S	S				Yes	2							
Temoporfin[224]	Photosensitizer							Yes	1							
Temozolomide[225]	Alkylanting agents							Yes	3 a 4							
Temsirolimus [226]	Immune-modulating agents	S					S↓	Yes	1							
Teniposide[227]	Antimitotic agents	S↓			↓	S		Yes	3 a 4							
Tioguanine[228]	Purine analogs							Yes	3 a 4							
Thiotepa[229]	Alkylanting agents	S						Yes	1							
Tivozanib[230]	Tyrosine kinase inhibitors	S	S					Yes	2	*	*	*	*	*	*	*
Tocilizumab[231]	Immune-modulating agents							Yes	1							
Topotecan[232]	Topoisomerase inhibitors	↑/↓					S	Yes	2							
Toremifene[233]	Hormonal agents	S					S	No	1					*		
Tositumomab[234]	Monoclonal antibodies							Yes	2							
Trabectedine[235]	Intercalating agents	S			S	S		Yes	3 a 4							
Tramadol[236]	Supportive care	S					S	No	No							
Trametinib[237]	Tyrosine kinase inhibitors			↓	↓		↓	Yes	3 a 4	*	*	*	*	*	*	*
Trastuzumab[238]	Monoclonal antibodies							Yes	1							

Trastuzumab emtansine[239]	Monoclonal antibodies	S↓					S	Yes	1	*	*	*	*	*	*	*
Treosulfan[240]	Alkylanting agents							Yes	3 a 4							
Tretinoin[241]	Retinoids	S		S	S			No	2							
Trifluridine and tipiracil[242]	Pyrimidine analogs							Yes	3 a 4							
Trioxide, Arsenic[243]	Cytotoxic	↓					S	Yes	3 a 4							
Triptorelin[244]	Hormonal agents							No	1							
Tropisetron[245]	Supportive care							No	No							
Valrubicin[246]	Anthracyclin/Anthracenediones	S					S	Yes	1							
Vandetanib[247]	Tyrosine kinase inhibitors	S					↓↓↓	No	2							
Vemurafenib[248]	Monoclonal antibodies	S↑	↓↓	↓	↓		S↓↓↓	No	2							
Venetoclax[249]	BH3 mimetics	S↓↓					S↓	Yes	1							
Vinblastine[250]	Antimitotic agents	S↓/↑					S↓/↑↑↑	Yes	1							
Vincristine[251]	Antimitotic agents	S↓					S↑/↓	Yes	1							
Vincristine, liposomal [252]	Antimitotic agents	S↓					S↑/↓	Yes	1							
Vindesine[253]	Antimitotic agents	S						Yes	1							
Vinflunine[254]	Antimitotic agents	S					S	Yes	1							
Vinorelbine[255]	Antimitotic agents	S↓					S↓	Yes	1					*		
Vorinostat[256]	HDAC inhibitors							Yes	2					*		

Ziv-aflibercept[257]	Monoclonal antibodies							Yes	1							

Notes: * Mechanism not associated with cytocrome P450 or transporters (e.g.: thrombocytopenia due to myelotoxicity), S, substrate, ↓ weak inhibition, ↓↓ moderate inhibition, ↓↓↓ strong inhibition, ↑ weak induction, ↑↑ moderate induction e ↑↑↑ strong induction. BH3, B-cell lymphoma 2 (Bcl-2) Homology 3 (BH3); CDK: cyclin-dependent kinase; HDAC, histone deacetilase, PARP, Poli-(ADP-ribose) polimerase. ** Levels of emetogenicity: 1 – minimum (<10% of patients), 2 – low (10-30%), 3-4 – moderate (30-90%) e 5 – high (>90%).

Case reports: Case reports or case series of fluctuations in international normalized ratio (INR) values and possible DDI have been described between warfarin and several anticancer drugs: 5-fluouracil[274-282], aminoglutethimide[283], aprepitant[284], azathioprine[285-291], cabozantinib[292], capecitabine[293-295], carboplatin[296], cisplatin[297], crizotinib[298], enzalutamide[299,300], erlotinib[301-303], exemestane[304], etoposide[296,305,306], geftinib[302,307], gemcitabine[294,308], ifosfamide combined with mesna[305,309], interferon[310], mercaptopurine[311], mitotane[312], paclitaxel[313], prednisolone[314], the R-ESHAP315 regimen (rituximab, etoposide, methylprednisolone, cytarabine and cisplatin), S1 (tegafur/gimeracil/oteracil)[316-318], sorafenib[319], tamoxifen[320-323], trastuzumab[324], vindesine[306], vismodegibe[325]. Although case reports demonstrate controversy about DDI between enzalutamide and warfarin, this hormonal agent has potential for interaction with DOACs[326]. There are two reports of clinically relevant non-major bleeding related to DOACs. In one of them, a 77-year-old male patient using apixaban for atrial fibrillation had bleeding in the popliteal fossa during treatment with cobimetinib and vemurafenib for metastatic melanoma[327]. In another case report, the association between an expansive mastectomy site hematoma and rivaroxaban use was considered as probable by a 67-year-old breast cancer patient who had been treated with four cycles of fluorouracil, epirubicin and cyclophosphamide (FEC), followed by four cycles of docetaxel, and also using letrozole[328]. Although none of these medications have clinically relevant DDI with DOACs, as this database review has shown, caution is recommended in combining them, especially in patients with an additional risk of bleeding, such as those with thrombocytopenia, renal or hepatic impairment, and certain types of cancers, such as luminal gastrointestinal tumors. Rare cases of major bleeds with DOACs[329] and LMWH[330] in cancer patients were also reported. The use of LMWH was associated with increased bleeding in a 56-year-old female patient receiving bevacizumab for non-small cell lung cancer[330]. A series of three cases[329] of acute hemorrhagic cardiac tamponade in cancer suggested an association between bleeding and DOACs in patients receiving nivolumab for lung carcinoma, experimental treatment with inhibitor PD-L1 (programmed death-ligand 1) due to tongue squamous cell carcinoma and ibrutinib for chronic lymphocytic leukemia. Although no mechanisms of DDI between these drugs and DOACs were described, the first two patients had neoplastic cells in the pericardial fluid and the last one used ibrutinib, that is associated with subclinical pericarditis.

No clinically significant DDI expected

Potentially weak DDI – dose adjustment or monitorization not required

Potentially clinically relevant DDI - dose adjustment or monitorization required

Coadministration not recommended

References

1. Bula Fauldfluor® FAULDF_V.13-19 e https://www.drugbank.ca/drugs/DB00544 e Gunes A, Coskun U, Boruban C, Gunel N, Babaoglu MO, Sencan O, Bozkurt A, Rane A, Hassan M, Zengil H, Yasar U: Inhibitory effect of 5-fluorouracil on cytochrome P450 2C9 activity in cancer patients. Basic Clin Pharmacol Toxicol. 2006 Feb;98(2):197-200.
2. Bula Verzenios® CDS14NOV18 e https://www.drugbank.ca/drugs/DB12001
3. Bula Zytiga® CCDS 1904 VPS 10 e https://www.drugbank.ca/drugs/DB05812
4. Bula Calquence® CAL002 e https://www.drugbank.ca/drugs/DB11703
5. Bula Megestat® Rev0416 e House L, Seminerio MJ, Mirkov S et al. Metabolism of megestrol acetate in vitro and the role of oxidative metabolites. Xenobiotica. 2018 Oct;48(10):973-983. e Fleming GF, Amato JM, Agresti M, et al. Megestrol acetate reverses multidrug resistance and interacts with P-glycoprotein. Cancer Chemother Pharmacol. 1992;29(6):445-9. e Wang L, Yang CP, Horwitz SB, Trail PA, Casazza AM.Reversal of the human and murine multidrug-resistance phenotype with megestrol acetate.Cancer Chemother Pharmacol. 1994;34(2):96-102.https://www.drugbank.ca/drugs/DB00351.
6. Bula Faudleuco® FLEU_V.7-15 e https://www.drugbank.ca/drugs/DB00650
7. Bula Bonviva® 0261247/19-0 e https://www.drugbank.ca/drugs/DB00710
8. Bula Zometa® CDS 07.10.16 VPS9 e https://www.drugbank.ca/drugs/DB00399

9. Bula Giotrif® 20190228/CR19-00 e https://www.drugbank.ca/drugs/DB08916
10. Bula Alecensa® CDS 6.0.A_Prof e https://www.drugbank.ca/drugs/DB11363
11. Bula Lemtrada® IB070319 e https://www.drugbank.ca/drugs/DB00087.
12. Bula Zyloric® Bula do profissional de saúde – Zyloric_BU_PS_003 CCDS v.18 e https://www.drugbank.ca/drugs/DB00437
13. Damia G, D'Incalci M: Clinical pharmacokinetics of altretamine. Clin Pharmacokinet. 1995;28(6):439-48 e https://www.drugbank.ca/drugs/DB00488.
14. Ethyol Prescribing Information (FDA) ID: 4102924 e https://www.drugbank.ca/drugs/DB01143
15. Lønning PE. Aminoglutethimide enzyme induction: pharmacological and endocrinological implications. Cancer Chemother Pharmacol. 1990;26(4):241-4 e https://www.drugbank.ca/drugs/DB00357.
16. Bula Arimidex® ARI011a e https://www.drugbank.ca/drugs/DB01217
17. Jurlina JL, Varcoe AR & Paxton JW: Pharmacokinetics of amsacrine in patients receiving combined chemotherapy for treatment of acute myelogenous leukemia. Cancer Chemother Pharmacol 1985; 14:21-25 e https://www.drugbank.ca/drugs/DB00276
18. Bula Tecentriq® V015_Prof_1200mg e https://www.drugbank.ca/drugs/DB11595
19. Bula Bavencio® 1062844/18-4 e https://www.drugbank.ca/drugs/DB11945
20. Bula Inlyta® LLD_Bra_CDSv7.0_20Nov2014_v9_INLCOR_10_VPS e https://www.drugbank.ca/drugs/DB06626
21. Bula Vidaza® 0752051/14-4 e https://www.drugbank.ca/drugs/DB00928
22. Bula Imuran® CCDS v25 e https://www.drugbank.ca/drugs/DB00993
23. Bula Imuran® CCDS v25 e https://www.drugbank.ca/drugs/DB00993
24. Bedeolaq Prescribing Information (FDA): https://www.accessdata.fda.gov/drugsatfda_docs/label/2014/206256lbl.pdf e https://www.drugbank.ca/drugs/DB05015
25. Bula Ribomustin® IPI 1710 V02 e https://www.drugbank.ca/drugs/DB06769
26. Bula Avastin® CDS 34.0_Prof e https://www.drugbank.ca/drugs/DB00112
27. Bula Targretin EMA (https://www.ema.europa.eu/en/documents/product-information/targretin-epar-product-information_pt.pdf). E https://www.drugbank.ca/drugs/DB00307
28. Bula Casodex® CAS011 e https://www.drugbank.ca/drugs/DB01128
29. Bula Tevableo®VPS - BU_06 e https://www.drugbank.ca/drugs/DB00290
30. Bula Blincyto BLI_PO_VPS_07-3 e https://www.drugbank.ca/drugs/DB09052
31. Bula Velcade® CCDS 1808 VPS09 e https://www.drugbank.ca/drugs/DB00188
32. Bula Bosulif® EMA (https://ec.europa.eu/health/documents/community-register/.../anx_125474_pt.pdf) e https://www.drugbank.ca/drugs/DB06616
33. Bula Adcetris® ADC_0815_1116_VPS e https://www.drugbank.ca/drugs/DB08870
34. Bula Suprefact® EMA: https://www.medicines.org.uk/emc/product/6807/smpc e https://www.drugbank.ca/drugs/DB06719

35. Bula Myleran® Myleran_COM_REV_NCDS01_L0681 e https://www.drugbank.ca/drugs/DB01008
36. Bula Jevtana® IB101116A e https://www.drugbank.ca/drugs/DB06772 e Paller CJ,Antonarakis ES. Cabazitaxel: a novel second-line treatment for metastatic castration-resistant prostate cancer Drug Design, Development and Therapy 2011:5 117–124
37. Bula Cometriq® EMA (https://www.ema.europa.eu/documents/.../cometriq-epar-product-information_pt.pdf) e https://www.drugbank.ca/drugs/DB08875
38. Asparlas® Prescribing Information (FDA): https://www.accessdata.fda.gov/drugsatfda_docs/label/2018/761102s000lbl.pdf e https://www.drugbank.ca/drugs/DB14730
39. Bula Xeloda® CDS 15.0D_Prof e https://www.drugbank.ca/drugs/DB01101
40. Bula Paraplatin® e https://www.drugbank.ca/drugs/DB00958
41. Bula Kypolis® KYP_SOL_VPS_03-1 e https://www.drugbank.ca/drugs/DB08889
42. Bula Becenun® e https://www.drugbank.ca/drugs/DB00262
43. Bula Libtayo®- FDA (https://www.accessdata.fda.gov/drugsatfda_docs/label/2018/761097s000lbl.pdf) e Markham A et al. Cemiplimab: First Global Approval. Drugs. 2018 Nov;78(17):1841-1846. e https://www.drugbank.ca/drugs/DB14707
44. Bula Zykadia® - EMA (https://www.ema.europa.eu/documents/.../zykadia-epar-product-information_pt.pdf) e https://www.drugbank.ca/drugs/DB09063
45. Bula Erbitux® 0252369/13-8 e https://www.drugbank.ca/drugs/DB00002
46. Bula Genuxal®0123844/15-2 e https://www.drugbank.ca/drugs/DB00531#targets
47. Bula Sandimmun® (CDS 05.08.14 2014-PSB/GLC-0689-s VPS3) e Parasrampuria et al. Br J Clin Pharmacol 2016;82:1591–1600. e https://www.drugbank.ca/drugs/DB00091#enzymes
48. Bula Androcur® VE0118 - CCDS – 10/12/13p e https://www.drugbank.ca/drugs/DB04839
49. Bula Cisplatina_VPS_06.2016 e https://www.drugbank.ca/drugs/DB00515
50. Bula Aracytin® LLD_Bra_CDSv10.0_22Jun2017_v3_ARAPOI_09_VPS e https://www.drugbank.ca/drugs/DB00987
51. Bula Leutastin® CCDS 1708 VPS 04 e https://www.drugbank.ca/drugs/DB00242
52. Bula Bonefós® VE0114-CCDS9 e https://www.drugbank.ca/drugs/DB00720
53. Bula Evoltra® EMA (https://www.ema.europa.eu/documents/.../evoltra-epar-product-information_pt.pdf) e https://www.drugbank.ca/drugs/DB00631
54. Bula Rivotril® CDS 7.0B_Prof e https://www.drugbank.ca/drugs/DB01068
55. Bula Leukeran® LEUKERAN_BU_PS_001 CCDS v17 e https://www.drugbank.ca/drugs/DB00291
56. Bula Cotellic® CDS 6.0F_Prof e Le Guern A et al. [Hemorrhagic syndrome during cobimetinib therapy: The need for caution in patients receiving new anticoagulant drugs].Ann Dermatol Venereol. 2017 May;144(5):383-384. e https://www.drugbank.ca/drugs/DB05239
57. Bula Codein® R_0199_01 e https://www.drugbank.ca/drugs/DB00318
58. Bula Xalkori® LLD_Bra_CDSv18.0_24Jun2016_v1_XALCAP_08_VPS e https://www.drugbank.ca/drugs/DB08865

59. Bula Tafinlar® VPS6 e https://www.drugbank.ca/drugs/DB08912
60. Vizimpro® Prescribing Information (FDA): https://www.accessdata.fda.gov/drugsatfda_docs/label/2018/211651s000lbl.pdf e https://www.drugbank.ca/drugs/DB11963
61. Bula Fauldacar® F.DACA_V.01-17 e https://www.drugbank.ca/drugs/DB00851
62. Bula Cosmegen® PX 638/X e https://www.drugbank.ca/drugs/DB00970
63. Bula Dalinvi® CCDS 1902 VPS 11.1 e https://www.drugbank.ca/drugs/DB09331
64. Bula Aranesp® ARA_SOL INJ_VPS_04-2 e https://www.drugbank.ca/drugs/DB00012
65. Zurth et al. Drug-drug interaction (DDI) of darolutamide with cytochrome P450 (CYP) and P-glycoprotein (P-gp) substrates: Results from clinical and in vitro studies. J Clin Oncol 2019; 37(7) Suppl:297-297.
66. Bula Sprycel SPRYCEL_VPS_v01_15032019 e https://www.drugbank.ca/drugs/DB01254
67. Bula Daunoblastina® LLD_Bra_CDSv3.0_21Nov2018_v1_DAUPOI_10_VPS e https://www.drugbank.ca/drugs/DB00694
68. Bula Caelyx® VPS 01 CCDS 1811 e https://www.drugbank.ca/drugs/DB00694
69. Bula Dacogen® CCDS 1810 VPS 08 e https://www.drugbank.ca/drugs/DB01262
70. Ontak® Prescribing information FDA: https://www.accessdata.fda.gov/drugsatfda_docs/label/2008/103767s5094lbl.pdf e https://www.drugbank.ca/drugs/DB00004
71. Bula Prolia® PRO_SOL_VPS_04-1 e https://www.drugbank.ca/drugs/DB06643
72. Bula Firmagon® BUL_FIR_SOL_INJ_VPS_02-1 e https://www.drugbank.ca/drugs/DB06699
73. Bula Decadron® Decadron injetável_BU_01_VPS e https://www.drugbank.ca/drugs/DB01234
74. Bula Cardioxane® BU_PS_349002.16 e https://www.drugbank.ca/drugs/DB00380
75. Bula Valium ® CDS 9.0_Prof e https://www.drugbank.ca/drugs/DB00829
76. Bula Difenidrin® AR_AM_12012017 e https://www.drugbank.ca/drugs/DB01075
77. Bula Dramin® DRCGM25_NSPC_0616_VPS, DR_NSPC_0619_VPS e CGM50_NSPC_0616_VPS e https://www.drugbank.ca/drugs/DB00985
78. Bula Unituxin® EMA: https://www.ema.europa.eu/en/documents/product-information/unituxin-epar-product-information_pt.pdf e https://www.drugbank.ca/drugs/DB09077
79. Bula Novalgina® IB220618A e Lampl C1, Likar R[Metamizole (dipyrone): mode of action, drug-drug interactions, and risk of agranulocytosis]. Schmerz. 2014 Dec;28(6):584-90 e https://www.drugbank.ca/drugs/DB04817
80. Bula Taxotere® IB110419 e https://www.drugbank.ca/drugs/DB01248
81. https://www.drugbank.ca/drugs/DB00757
82. Bula Adriblastina RD® 0125540/19-1 e https://www.drugbank.ca/drugs/DB00997
83. Bula Doxopeg® 0439330/15-9

84. Marinol® Prescribing Information (FDA): https://www.accessdata.fda.gov/drugsatfda_docs/label/2017/018651s029lbl.pdf e https://www.drugbank.ca/drugs/DB00470
85. Bula Imfinzi® IMF006 e https://www.drugbank.ca/drugs/DB11714
86. Bula Xtandi® 198568-XTA-BRA e https://www.drugbank.ca/drugs/DB08899
87. Bula Farmorubicina CS® LLD_Bra_CDSv9.0_21Nov2018_v1_FCSSOI_11_VPS e https://www.drugbank.ca/drugs/DB00445
88. Bula Eprex® CCDS 1708 VPS 08.2 e https://www.drugbank.ca/drugs/DB00016
89. Bula Empliciti® Rev0818 e https://www.drugbank.ca/drugs/DB06317
90. Balversa® Prescribing Information (FDA): https://www.accessdata.fda.gov/drugsatfda_docs/label/2019/212018s000lbl.pdf e https://www.drugbank.ca/drugs/DB12147 e https://www.drugbank.ca/drugs/DB12147
91. Bula Halaven® CCDS V.12 JUN2018 e https://www.drugbank.ca/drugs/DB08871
92. Bula Tarceva® CDS 17.0C_Prof e https://www.drugbank.ca/drugs/DB00530
93. Zanosar®, Streptozocin,https://www.drugbank.ca/drugs/DB00428
94. Bula Tevaetopo® BU_06 e https://www.drugbank.ca/drugs/DB00773
95. Emcyt®: http://labeling.pfizer.com/showlabeling.aspx?id=671 e https://www.drugbank.ca/drugs/DB01196
96. Bula Afinitor® VPS17 = Afinitor_Bula_Profissional e https://www.drugbank.ca/drugs/DB01590
97. Bula Aromasin® LLD_Bra_CDSv10.0_07Sep2017_v2_AROCOR_14_VPS e https://www.drugbank.ca/drugs/DB00990
98. Bula Durogesic® CCDS 1812 VPS 09 e https://www.drugbank.ca/drugs/DB00813
99. Bula Granulokine®GRA_SOLINJ_VPS_08-3 e https://www.drugbank.ca/drugs/DB00099
100. https://www.drugbank.ca/drugs/DB00322
101. Bula Fludalibbs® FLUD_V.11-17 e https://www.drugbank.ca/drugs/DB01073
102. Bula Flutamida 7003408-01 e https://www.drugbank.ca/drugs/DB00499
103. Bula Emend Injetável® EMEND INJETÁVEL_BU09_052018_VPS e https://www.drugbank.ca/drugs/DB06717
104. Bula Muphoran® http://www.anvisa.gov.br/datavisa/fila_bula/frmVisualizarBula.asp?pNuTransacao=21044862017&pIdAnexo=9898843 e https://www.drugbank.ca/drugs/DB04106
105. Bula Faslodex® FAS022 e https://www.drugbank.ca/drugs/DB00947
106. Bula Iressa® IRE011 e Hiraide M, Minowa Y, Nakano Y, et al. Drug interactions between tyrosine kinase inhibitors (gefitinib and erlotinib) and warfarin: Assessment of international normalized ratio elevation characteristics and in vitro CYP2C9 activity.J Oncol Pharm Pract. 2018 Sep 26:1078155218801061. e https://www.drugbank.ca/drugs/DB00317
107. Bula Gencitabina Cloridrato de Gencitabina_VPS_01.2019 e https://www.drugbank.ca/drugs/DB00441
108. Bula Mylotarg® EMA: https://www.ema.europa.eu/en/documents/product-information/mylotarg-epar-product-information_pt.pdf e https://www.drugbank.ca/drugs/DB00056

109. Xospata® Prescribing Information (FDA): https://www.accessdata.fda.gov/drugsatfda_docs/label/2018/211349s000lbl.pdf e https://www.drugbank.ca/drugs/DB12141
110. Daurismo® Prescribing Information (FDA): https://www.accessdata.fda.gov/drugsatfda_docs/label/2018/210656s000lbl.pdf e https://www.drugbank.ca/drugs/DB11978
111. Bula Kytril® 0268524/18-8 e https://www.drugbank.ca/drugs/DB00889
112. Bula Zoladex® ZOL_ZOL_LA012a e https://www.drugbank.ca/drugs/DB00014
113. Bula Cortisonal® (succinato sódico de hidrocortisona) 0695176/14-7 e https://www.drugbank.ca/drugs/DB00741
114. Dilaudid Prescribing Information https://www.accessdata.fda.gov/drugsatfda_docs/label/2016/019891s024,019892s029lbl.pdf e https://www.drugbank.ca/drugs/DB00327
115. Bula Hydrea® Rev1218 e https://www.drugbank.ca/drugs/DB01005
116. Bula Zevalin® https://www.ema.europa.eu/en/documents/product-information/zevalin-epar-product-information_pt.pdf e https://www.drugbank.ca/drugs/DB00078
117. Bula Imbruvica® CCDS 1905 VPS 25 e https://www.drugbank.ca/drugs/DB09053
118. Bula Zavedos® LLD_Bra_CDSv5.0_21Nov2018_v1_ZAVPOI_09 e https://www.drugbank.ca/drugs/DB01177
119. Bula Zydelig® https://www.ema.europa.eu/en/documents/product-information/zydelig-epar-product-information_en.pdf e https://www.drugbank.ca/drugs/DB09054
120. Bula Holoxane® 0287146/13-7 e https://www.drugbank.ca/drugs/DB01181
121. Bula Glivec® VPS21= Glivec_Bula_Profissional e https://www.drugbank.ca/drugs/DB00619
122. Bula Benponsa® EMA https://www.ema.europa.eu/en/documents/product-information/besponsa-epar-product-information_pt.pdf e https://www.drugbank.ca/drugs/DB05889
123. Bula Roferon-A® CDS 7.0B_Prof e Brennan BJ, Xu ZX, Grippo JF.Effect of peginterferon alfa-2a (40KD) on cytochrome P450 isoenzyme activity. Br J Clin Pharmacol. 2013;75(2):497-506 e https://www.drugbank.ca/drugs/DB05258
124. Bula Alfainterferona 2b recombinante Aché 0070762/19-7 e https://www.drugbank.ca/drugs/DB00105
125. Bula Pegasys® CDS 18.0B_Prof e https://www.drugbank.ca/drugs/DB00034
126. Bula Pegintron® PEGINTRON MELANOMA_BU12_092017_VPS e https://www.drugbank.ca/drugs/DB00105
127. Bula Proleukin® BU 01 PS - 349014. 13 e https://www.drugbank.ca/drugs/DB00041
128. Bula Yervoy® Rev0515 e https://www.drugbank.ca/drugs/DB06186
129. Bula Camptosar® LLD_Bra_CDSv14.0_19Jul2018_v1_CAMSUI_13_VPS e https://www.drugbank.ca/drugs/DB00762
130. Bula Onivyde® EMA https://www.ema.europa.eu/en/documents/product-information/onivyde-epar-product-information_pt.pdf
131. Ixempra Prescribing Information (FDA): https://www.accessdata.fda.gov/drugsatfda_docs/label/2011/022065s006lbl.pdf e https://www.drugbank.ca/drugs/DB04845
132. Bula Ninlaro® NI_CAPS_1215_1217_VPS e https://www.drugbank.ca/drugs/DB09570

133. Bula Tykerb® CDS 17.04.19 VPS4 e https://www.drugbank.ca/drugs/DB01259
134. Bula Oncaspar® 0263668/19-9 e https://www.drugbank.ca/drugs/DB00023
135. Bula Vitrakvi FDA (FDA prescribing information ID: 4354331): https://www.accessdata.fda.gov/drugsatfda_docs/label/2018/211710s000lbl.pdf e https://www.drugbank.ca/drugs/DB14723
136. Somatuline Autogel®1052991/18-8 e https://www.drugbank.ca/drugs/DB06791
137. Bula Revlimid® CCDS 21_Prof e https://www.drugbank.ca/drugs/DB00480
138. Bula Lenvima® CCDS_V09 Apr2018 e https://www.drugbank.ca/drugs/DB09078
139. Bula Femara® CDS 15.12.16 2016-PSB/GLC-0857-s VPS5 e https://www.drugbank.ca/drugs/DB01006
140. Bula Lupron® 0479700/13-1 e https://www.drugbank.ca/drugs/DB00007
141. Lenograstin https://www.drugbank.ca/drugs/DB13144
142. Khapzory® Prescribing Information (FDA): https://www.accessdata.fda.gov/drugsatfda_docs/label/2018/211226s000lbl.pdf e https://www.drugbank.ca/drugs/DB11596
143. Bula Citostal® Rev0815 e https://www.drugbank.ca/drugs/DB01206
144. Bula Lorax® LLD_Bra_CDSv6.0_02Aug2018_v1_LRXCOM_07_VPS e https://www.drugbank.ca/drugs/DB00186
145. Luthatera® Prescribing Information (FDA): https://www.accessdata.fda.gov/drugsatfda_docs/label/2018/208700s000lbl.pdf e https://www.drugbank.ca/drugs/DB13985
146. https://www.drugbank.ca/drugs/DB00888
147. Bula Alkeran® Bula do profissional de saúde – Cód BU_PS_alkeran_00-17 150.4.04.12-01 e https://www.drugbank.ca/drugs/DB01042
148. Bula Purinethol® 93.1.08.11-01 Puri_BU_PS_01.16 [Excella&GSK] e https://www.drugbank.ca/drugs/DB01033
149. Bula Mitexan® 0125442/15-1 e https://www.drugbank.ca/drugs/DB09110
150. Bula Mytedom® AR_14032017_0405761179 e https://www.drugbank.ca/drugs/DB00333
151. Succinato de Metilprednisolona Novafarma Bula ME – 20001204V02 e https://www.drugbank.ca/drugs/DB00959
152. Bula Plasil® IB240817A e https://www.drugbank.ca/drugs/DB01233
153. Bula Fauldmetro® FMET_v.15-17 e https://www.drugbank.ca/drugs/DB00563
154. Bula Dormonid® CDS 7.0D_Prof_INJ e CDS 5.0D_Prof_COM e https://www.drugbank.ca/drugs/DB00683
155. Bula Rydapt® VPS1 = Rydapt_Bula_Profissional e https://www.drugbank.ca/drugs/DB06595
156. Bula Mepact® EMA https://www.ema.europa.eu/en/documents/product-information/mepact-epar-product-information_pt.pdf e https://www.drugbank.ca/drugs/DB13615
157. Mytomicin https://www.drugbank.ca/drugs/DB00305
158. Bula Lisodren® EMA https://www.ema.europa.eu/en/documents/product-information/lysodren-epar-product-information_pt.pdf e https://www.drugbank.ca/drugs/DB00648

159. Bula Evomixan® 1210LV001 B50007012/00 e https://www.drugbank.ca/drugs/DB01204
160. Bula Dimorf® R_M_0097_0363_02 e https://www.drugbank.ca/drugs/DB00295
161. Cesamet Prescribing Information (FDA): https://www.accessdata.fda.gov/drugsatfda_docs/label/2006/018677s011lbl.pdfe https://www.drugbank.ca/drugs/DB00486
162. Bula Abraxane® CCDS 15_Prof e https://www.drugbank.ca/drugs/DB01229 e Yu K, Mantha S, Tjan C et al. A pilot study of gemcitabine, nab-paclitaxel, PEGPH20 (PAG) and rivaroxaban for advanced pancreatic adenocarcinoma: Interim safety and efficacy analysis. Annals of Oncology 2018; 29 Suppl 8: mdy282.113
163. Bula Atriance® EMA https://ec.europa.eu/health/documents/community-register/2017/20170616137949/anx_137949_pt.pdf e https://www.drugbank.ca/drugs/DB01280
164. Bula Nerlynx® FDA https://www.accessdata.fda.gov/drugsatfda_docs/label/2017/208051s000lbl.pdf e https://www.drugbank.ca/drugs/DB11828
165. Bula Tasigna® CDS 30.01.18 2017-PSB/GLC-0916-s VPS15 e https://www.drugbank.ca/drugs/DB04868
166. Anandron® Prescribing Information (FDA): https://www.accessdata.fda.gov/drugsatfda_docs/label/2016/207631Orig1s000lbl.pdf
167. Bula Cimaher® CIMAher_VPS_V5 e https://www.drugbank.ca/drugs/DB06192
168. Bula OFEV® 20180427/ CM19-00 e https://www.drugbank.ca/drugs/DB09079
169. Bula Zejula® EMA https://www.ema.europa.eu/en/documents/product-information/zejula-epar-product-information_pt.pdf e https://www.drugbank.ca/drugs/DB11793
170. Bula Opdivo® OPDIVO_VPS_v01_06122018 e https://www.drugbank.ca/drugs/DB09035
171. Bula Gazyva® CDS 7.0A_Prof e https://www.drugbank.ca/drugs/DB08935
172. Bula Sandostatin® VPS7 = Sandostatin_Bula_Profissional e https://www.drugbank.ca/drugs/DB00104
173. Bula Arzerra® VPS2 = Arzerra_Bula_Profissional e https://www.drugbank.ca/drugs/DB06650
174. Bula Lynparza® EMA https://ec.europa.eu/health/documents/community-register/.../anx_130230_pt.pdf e https://www.drugbank.ca/drugs/DB09074
175. Bula Lartruvo® 0286837/18-7 e https://www.drugbank.ca/drugs/DB06043
176. Bula Omeprazol sódico endovenoso omeprazol_sódico_V3_VPS e https://www.drugbank.ca/drugs/DB00338
177. Bula Zofran® VPS2 = Zofran_Bula_Profissional. e Sanwald P ,David M, Dow J. Characterization of the cytochrome P450 enzymes involved in the in vitro metabolism of dolasetron. Comparison with other indole-containing 5-HT3 antagonists. Drug Metab. Dispos 1996; 24, 602-609. e https://www.drugbank.ca/drugs/DB00904
178. Bula Tagrisso® TAG009 e https://www.drugbank.ca/drugs/DB09330
179. Bula Oxalibbs® OXAL_v.11-19. e https://www.drugbank.ca/drugs/DB00526
180. Bula Oxycontin® VE0219-CCDS13 e https://www.drugbank.ca/drugs/DB00497
181. Bula Taxol® Rev0916 e https://www.drugbank.ca/drugs/DB01229

182. Bula Ibrance® LLD_Bra_CDSv12.0_20Mar2019_v1_IBRCAP_11_VPS e https://www.drugbank.ca/drugs/DB09073
183. Bula Palonosetrona Accord VPS_04.2019 e https://www.drugbank.ca/drugs/DB00377
184. Bula Akynzeo® VE0219 e Akynzeo Prescribing Information FDA: https://www.accessdata.fda.gov/drugsatfda_docs/label/2018/210493s000lbl.pdf e https://www.drugbank.ca/drugs/DB14019 e https://www.drugbank.ca/drugs/DB09048
185. Bula Pamidron® AR_24072017 e https://www.drugbank.ca/drugs/DB00282
186. Bula Vectibix® VEC_SOL_VPS_04-4 e https://www.drugbank.ca/drugs/DB01269
187. Bula Farydak EMA https://www.ema.europa.eu/documents/.../farydak-epar-product-information_pt.pdf e https://www.drugbank.ca/drugs/DB06603
188. Bula Tylenol® 0191653/18-0 e https://www.drugbank.ca/drugs/DB00316
189. Bula Votrient® CDS 18.08.17 2017-PSB/GLC-0891-s VPS2 e https://www.drugbank.ca/drugs/DB06589
190. Bula Keytruda® KEYTRUDA_BU62_102018_VPS e https://www.drugbank.ca/drugs/DB09037
191. Bula Alimta® 1076781/15-9 e https://www.drugbank.ca/drugs/DB00642
192. https://www.drugbank.ca/drugs/DB00552
193. Bula Perjeta® CDS 10.0B_Prof e https://www.drugbank.ca/drugs/DB06366
194. Bula Pixuvri® https://www.ema.europa.eu/en/documents/product-information/pixuvri-epar-product-information_pt.pdf e https://www.drugbank.ca/drugs/DB06193
195. Bula Imnovid EMA https://www.ema.europa.eu/en/documents/product-information/imnovid-epar-product-information_pt.pdf e https://www.drugbank.ca/drugs/DB08910
196. Bula Iclusig® ICL_BR01_EMA_MAR_2018 e https://www.drugbank.ca/drugs/DB08901
197. Folotyn Prescribing Information (FDA): https://www.accessdata.fda.gov/drugsatfda_docs/label/2009/022468lbl.pdf e https://www.drugbank.ca/drugs/DB06813
198. Bula Meticorten® METICORTEN_BU09_082017_VPS e https://www.drugbank.ca/drugs/DB00635
199. Bula PredYes® 0296805/18-3 e https://www.drugbank.ca/drugs/DB00860
200. Procarbazine: https://www.drugbank.ca/drugs/DB01168
201. Prochlorperazine: https://www.drugbank.ca/drugs/DB00433
202. Bula Xofigo® EMA https://ec.europa.eu/health/documents/community-register/2017/20170814138527/anx_138527_pt.pdf e https://www.drugbank.ca/drugs/DB08913
203. Bula Evista® 1144858/16-0 e https://www.drugbank.ca/drugs/DB00481
204. Raltitrexed: https://www.drugbank.ca/drugs/DB00293
205. Bula Cyramza® 0515900/19-8 e https://www.drugbank.ca/drugs/DB05578
206. Bula Ranitidina 1177969/16-1 e https://www.drugbank.ca/drugs/DB00863

207. Bula Stivarga® VE0118-CCDS10 e https://www.drugbank.ca/drugs/DB08896
208. Bula Kisqali® SmPC v2.0 VPS2 e https://www.drugbank.ca/drugs/DB11730
209. Bula Mabthera® CDS 28.0C_Prof _SC e https://www.drugbank.ca/drugs/DB00073
210. Varuby Prescribing Information (FDA): https://www.ema.europa.eu/en/documents/product-information/varuby-epar-product-information_en.pdf e https://www.drugbank.ca/drugs/DB09291
211. Istodax® Prescribing Information (FDA): https://www.accessdata.fda.gov/drugsatfda_docs/label/2009/022393lbl.pdf e https://www.drugbank.ca/drugs/DB06176
212. Bula Rubraca® EMA: https://www.ema.europa.eu/en/documents/product-information/rubraca-epar-product-information_pt.pdf e https://www.drugbank.ca/drugs/DB12332
213. Bula Jakavi® CDS 16.04.18 2018-PSB/GLC-0930-s VPS5 e https://www.drugbank.ca/drugs/DB08877
214. Bula Noripurum® NOEV_1215_0217_VPS e https://www.drugbank.ca/drugs/DB09146
215. Leukine® Prescribing Information (FDA) https://www.accessdata.fda.gov/drugsatfda_docs/label/2017/103362s5237lbl.pdf e https://www.drugbank.ca/drugs/DB00020
216. Bula Rapamune® LLD_Bra_CDSv40.0_12May2017_v4_RPMDRG_17_VPS e https://www.drugbank.ca/drugs/DB00877
217. Bula Nexavar® VE0115-CCDS21 e https://www.drugbank.ca/drugs/DB00398
218. Bula Sutent® LLD_Bra_CDSv40.0_07Maio2018_v2_SYNCAP_25_VPS e https://www.drugbank.ca/drugs/DB01268
219. Bula Prograf® 0444247/19-4 e https://www.drugbank.ca/drugs/DB00864
220. Talzenna® Prescribing Information (FDA): https://www.accessdata.fda.gov/drugsatfda_docs/label/2018/211651s000lbl.pdf e https://www.drugbank.ca/drugs/DB11760
221. Bula Funed Talidomida BULFM-0041-REV07_Profissional e https://www.drugbank.ca/drugs/DB01041
222. Bula Novaldex® NOL+NOLD008 e https://www.drugbank.ca/drugs/DB00675
223. Bula Teysuno® EMA: https://www.ema.europa.eu/en/documents/product-information/teysuno-epar-product-information_en.pdf e https://www.drugbank.ca/drugs/DB09256 e https://www.drugbank.ca/drugs/DB09257 e https://www.drugbank.ca/drugs/DB03209
224. Bula Foscan® EMA: https://www.ema.europa.eu/en/documents/product-information/foscan-epar-product-information_en.pdf e https://www.drugbank.ca/drugs/DB11630
225. Bula Temodal ® TEMODAL_BU15_012017_VPS e https://www.drugbank.ca/drugs/DB00853
226. Bula Torisel® LLD_Bra_CDSv23.0_16Aug2016_v6_TRSSOI_17_VPS e https://www.drugbank.ca/drugs/DB06287
227. Vumon® Prescribing Information (FDA): https://www.accessdata.fda.gov/drugsatfda_docs/label/2011/020119s010s011lbl.pdf e https://www.drugbank.ca/drugs/DB00444
228. Bula Lanvis® Lanvis_BU_PS_01.16 e https://www.drugbank.ca/drugs/DB00352
229. Bula Tepadina® EMA https://www.ema.europa.eu/en/documents/product-information/tepadina-epar-product-information_pt.pdf e Thiotepa: https://www.drugbank.ca/drugs/DB04572

230. Bula Fotivda® https://www.ema.europa.eu/en/documents/product-information/fotivda-epar-product-information_en.pdf e https://www.drugbank.ca/drugs/DB11800
231. Bula Actemra® CDS 18.0_Prof e https://www.drugbank.ca/drugs/DB06273
232. Bula Hycamtin® CDS 10.09.18 2018-PSB/GLC-0941-s VPS3 e https://www.drugbank.ca/drugs/DB01030
233. Fareston® Prescribing Information (FDA): https://www.accessdata.fda.gov/drugsatfda_docs/label/2011/020497s006lbl.pdf e https://www.drugbank.ca/drugs/DB00539
234. https://www.drugbank.ca/drugs/DB00081
235. Bula Yondelis® EMA https://www.ema.europa.eu/en/documents/product-information/yondelis-epar-product-information_pt.pdf e Trabectedine https://www.drugbank.ca/drugs/DB05109
236. Bula Tramal® e https://www.drugbank.ca/drugs/DB00193
237. Bula Mekinist® CDS 12.11.18 2018-PSB/GLC-0950-s VPS7 e https://www.drugbank.ca/drugs/DB08911
238. Bula Herceptin® SC CDS 19.0A_Prof_IV e https://www.drugbank.ca/drugs/DB00072
239. Bula Kadcyla® CDS 8.0_Prof e https://www.drugbank.ca/drugs/DB05773
240. Treosulfan. http://www.bccancer.bc.ca/drug-database-site/Drug%20Index/Treosulfaninterim_monograph_14Jul2011.pdf e Treosulfan https://www.drugbank.ca/drugs/DB11678
241. Bula Versanoid® Vesanoid_AR030419_Profissional de saúde e https://www.drugbank.ca/drugs/DB00755
242. Bula Lonsurf® EMA: https://www.ema.europa.eu/documents/.../lonsurf-epar-product-information_pt.pdf e https://www.drugbank.ca/drugs/DB00432 e https://www.drugbank.ca/drugs/DB09343
243. Trisenox® BU_02 e https://www.drugbank.ca/drugs/DB01169
244. Bula Gonapeptyl Depot® CCDS 2011/11 V01 e https://www.drugbank.ca/drugs/DB06825
245. https://www.drugbank.ca/drugs/DB11699
246. Valstar® Prescribing Information (FDA): https://www.accessdata.fda.gov/drugsatfda_docs/label/2016/020892s019lbl.pdf e https://www.drugbank.ca/drugs/DB00385
247. Bula Caprelsa® IB210417 e https://www.drugbank.ca/drugs/DB05294
248. Bula Zelboraf® CDS 9.0C_Prof e https://www.drugbank.ca/drugs/DB08881
249. Bula Venclexta® BU07 e https://www.drugbank.ca/drugs/DB11581
250. Bula Velban® 0513781/14-1 e https://www.drugbank.ca/drugs/DB00570
251. Bula Sulfato de Vincristina Accord Sulfato de Vincristina_VPS_05.2016 e https://www.drugbank.ca/drugs/DB00541
252. Marqibo® Prescribing Information (FDA) 1.14 Labeling
253. Vindesine: https://www.drugbank.ca/drugs/DB00309 e https://www.medicines.org.uk/emc/files/pil.5266.pdf
254. Bula Javlor® 1921634/16-3 e https://www.drugbank.ca/drugs/DB11641

255. Bula Navelbine® Navelbine IV – Bula do Profissional de Saúde – Versão 03 – Jan 2019 e https://www.drugbank.ca/drugs/DB00361.
256. Zolinza® Prescribing Information (FDA): https://www.accessdata.fda.gov/drugsatfda_docs/label/2011/021991s002lbl.pdf e https://www.drugbank.ca/drugs/DB02546
257. Bula Zaltrap® IB101116 e https://www.drugbank.ca/drugs/DB08885
258. Bula Eliquis® LLD_Bra_CCDS_17Nov2017_v2_ELICOR_18_VPS e https://www.drugbank.ca/drugs/DB06605
259. Bula Pradaxa® 20150414/ C15-01 e https://www.drugbank.ca/drugs/DB06695
260. Bula Lixiana® LIX_COM_ VPS_06_7 e https://www.drugbank.ca/drugs/DB09075
261. Bula Xarelto® VE0219-CCDS14 e VE0219-CCDS11 e https://www.drugbank.ca/drugs/DB06228
262. Bula Marevan® AR030914 e https://www.drugbank.ca/drugs/DB00682
263. Bula Clexane® IB100214 e https://www.drugbank.ca/drugs/DB01225
264. Bula Arixtra® Arixtra_BU_PS_001 e 197.2.05.16-00 e https://www.drugbank.ca/drugs/DB00569
265. U.S. Food and Drug Administration. Drug Development and Drug Interactions: Table of Substrates, Inhibitors and Inducers https://www.fda.gov/drugs/drug-interactions-labeling/drug-development-and-drug-interactions-table-substrates-inhibitors-and-inducers
266. University of Washington. https://depts.washington.edu/anticoag
267. Cancer Drug Interactions. https://cancer-druginteractions.org/
268. Steffel J, Verhamme P, Potpara TS,et al. The 2018 European Heart Rhythm Association Practical Guide on the use of non-vitamin K antagonist oral anticoagulants in patients with atrial fibrillation. Eur Heart J. 2018;39(16):1330-1393.
269. Short NJ, Connors JM. New oral anticoagulants and the cancer patient. Oncologist 2014;19:82–93.
270. Mescape. https://reference.medscape.com/drug-interactionchecker
271. SuperCYP: http://bioinformatics.charite.de/supercyp
272. Lexicomp UptoDate. https://www.uptodate.com/home/drugs-drug-interaction
273. Drugs.com. https://www.drugs.com/interaction/list/
274. Kolesar JM, Johnson CL, Freeberg BL, Berlin JD, Schiller JH. Warfarin-5-FU interaction--a consecutive case series. Pharmacotherapy. 1999;19(12):1445-9.
275. Masci G, Magagnoli M, Zucali PA, Castagna L, Carnaghi C, Sarina B, Pedicini V, Fallini M, Santoro A. Minidose warfarin prophylaxis for catheter-associated thrombosis in cancer patients: can it be safely associated with fluorouracil-based chemotherapy? J Clin Oncol. 2003;21(4):736-9.
276. Davis DA, Fugate SE. Increasing warfarin dosage reductions associated with concurrent warfarin and repeated cycles of 5-Fluorouracil therapy. Pharmacotherapy. 2005;25(3):442-7.

277. Morita N, Kashihara K, Tagashira H, Otsuka H, Yoneda K, Murase T, Tsujikawa T, Furutani S, Furutani K, Minato M, Nishitani H. Two cases of retroperitoneal hematoma caused by combination of anticoagulant therapy and 5-fluorouracil. J Med Invest. 2005;52(1-2):114-7.
278. Brown MC. Multisite mucous membrane bleeding due to a possible interaction between warfarin and 5-fluorouracil. Pharmacotherapy. 1997;17(3):631-3.
279. Brown MC. An adverse interaction between warfarin and 5-fluorouracil: A case report and review of the literature. Chemotherapy. 1999;45(5):392-5.
280. Brown MC. Interaction between warfarin and 5-fluorouracil, not between warfarin and levamisole. Clin Pharmacol Ther. 1998;64(2):233.
281. Aki Z, Kotiloğlu G, Ozyilkan O. A patient with a prolonged prothrombin time due to an adverse interaction between 5-fluorouracil and warfarin. Am J Gastroenterol. 2000;95(4):1093-4.
282. Carabino J, Wang F. International Normalized Ratio fluctuation with warfarin-fluorouracil therapy. Am J Health Syst Pharm. 2002;59(9):875.
283. Lønning PE, Kvinnsland S, Jahren G. Aminoglutethimide and warfarin. A new important drug interaction. Cancer Chemother Pharmacol. 1984;12(1):10-2.
284. Ohno Y, Yamada M, Yamaguchi R, Hisaka A, Suzuki H. Persistent drug interaction between aprepitant and warfarin in patients receiving anticancer chemotherapy. Int J Clin Pharm. 2014;36(6):1134-7.
285. Rivier G, Khamashta MA, Hughes GR. Warfarin and azathioprine: a drug interaction does exist. Am J Med. 1993;95(3):342.
286. Singleton JD, Conyers L. Warfarin and azathioprine: an important drug interaction. Am J Med. 1992;92(2):217.
287. Pushpakom SP, Gambhir N, Latif A, Hadfield KD, Campbell S, Newman WG. Exacerbation of hereditary warfarin resistance by azathioprine. Clin Appl Thromb Hemost. 2011;17(3):293-6.
288. Vazquez SR, Rondina MT, Pendleton RC. Azathioprine-induced warfarin resistance. Ann Pharmacother. 2008;42(7):1118-23.
289. Ng HJ, Crowther MA. Azathioprine and inhibition of the anticoagulant effect of warfarin: evidence from a case report and a literature review. Am J Geriatr Pharmacother. 2006;4(1):75-7.
290. Walker J, Mendelson H, McClure A, Smith MD. Warfarin and azathioprine: clinically significant drug interaction. J Rheumatol. 2002;29(2):398-9.
291. Havrda DE, Rathbun S, Scheid D. A case report of warfarin resistance due to azathioprine and review of the literature. Pharmacotherapy. 2001;21(3):355-7.
292. Foxx-Lupo WT, Sing S, Alwan L, Tykodi SS. A Drug Interaction Between Cabozantinib and Warfarin in a Patient With Renal Cell Carcinoma. Clin Genitourin Cancer. 2016; 14(1):e119-21.
293. Yildirim Y, Ozyilkan O, Akcali Z, Basturk B. Drug interaction between capecitabine and warfarin: a case report and review of the literature. Int J Clin Pharmacol Ther. 2006; 44(2):80-2.

294. Saif MW, Wasif N. Interaction between capecitabine and gemcitabine with warfarin in a patient with pancreatic cancer. JOP. 2008;9(6):739-43.
295. Copur MS, Ledakis P, Bolton M, Morse AK, Werner T, Norvell M, Muhvic J, Chu E. An adverse interaction between warfarin and capecitabine: a case report and review of the literature. Clin Colorectal Cancer. 2001;1(3):182-4.
296. Le AT, Hasson NK, Lum BL. Enhancement of warfarin response in a patient receiving etoposide and carboplatin chemotherapy. Ann Pharmacother. 1997;31(9):1006-8.
297. Yano R, Kurokawa T, Tsuyoshi H, Shinagawa A, Sawamura Y, Matsunaga A, Nakamura T, Yoshida Y, Yoneda M, Kotsuji F, Masada M. Transient elevation of international normalized ratio during cisplatin-based chemotherapy in patients who are taking warfarin. Ann Pharmacother. 2011;45(10):e55.
298. Kubomura Y, Ise Y, Wako T, Katayama S, Noro R, Kubota K. A Drug Interaction between Crizotinib and Warfarin in Non-Small-Cell Lung Cancer: A Case Report. J Nippon Med Sch. 2017;84(6):291-293.
299. Casserly EA, Rogers SE, Keisner SV. Lack of interaction between enzalutamide and warfarin in a metastatic castration-resistant prostate cancer patient. J Oncol Pharm Pract. 2017;23(1):68-70.
300. Parrett JL, Reaves AB, Self TH, Owens RE. Enzalutamide-warfarin interaction necessitating warfarin dosage adjustment: A case report of successful clinical management. J Clin Pharm Ther. 2018;43(2):276-279.
301. Thomas KS, Billingsley A, Amarshi N, Nair BA. Elevated international normalized ratio associated with concomitant warfarin and erlotinib. Am J Health Syst Pharm. 2010 Sep 1;67(17):1426-9.
302. Hiraide M, Minowa Y, Nakano Y, Suzuki K, Shiga T, Nishio M, Miyoshi J, Takahashi H, Hama T. Drug interactions between tyrosine kinase inhibitors (gefitinib and erlotinib) and warfarin: Assessment of international normalized ratio elevation characteristics and in vitro CYP2C9 activity. J Oncol Pharm Pract. 2018:1078155218801061.
303. Thomas KS, Billingsley A, Amarshi N, Nair BA. Elevated international normalized ratio associated with concomitant warfarin and erlotinib. Am J Health Syst Pharm. 2010;67(17):1426-9.
304. Dush A, Williams A, Mangini N, Klaczak K. Initiation of exemestane in two warfarin-treated patients leading to elevation and variability of INR. J Oncol Pharm Pract. 2016;22(2):371-3.
305. Okada N, Watanabe H, Kagami S, Ishizawa K. Ifosfamide and etoposide chemotherapy may interact with warfarin, enhancing the warfarin induced anticoagulant response. Int J Clin Pharmacol Ther. 2016;54(1):58-61.
306. Ward K, Bitran JD. Warfarin, etoposide, and vindesine interactions. Cancer Treat Rep. 1984;68(5):817-8.
307. Onoda S, Mitsufuji H, Yanase N, Ryuge S, Kato E, Wada M, Ishii K, Hagiri S, Yamamoto M, Yokoba M, Yanaihara T, Kuboto M, Takada N, Katagiri M, Abe T, Tanaka N, Kobayashi H, Masuda N. Drug interaction between gefitinib and warfarin. Jpn J Clin Oncol. 2005;35(8):478-82.
308. Kinikar SA, Kolesar JM. Identification of a gemcitabine-warfarin interaction. Pharmacotherapy. 1999;19(11):1331-3.

309. Hall G, Lind MJ, Huang M, Moore A, Gane A, Roberts JT, Cantwell BM. Intravenous infusions of ifosfamide/mesna and perturbation of warfarin anticoagulant control. Postgrad Med J. 1990;66(780):860-1.
310. Adachi Y, Yokoyama Y, Nanno T, Yamamoto T. Potentiation of warfarin by interferon. BMJ. 1995;311(7000):292.
311. Martin LA, Mehta SD. Diminished anticoagulant effects of warfarin with concomitant mercaptopurine therapy. Pharmacotherapy. 2003;23(2):260-4.
312. Cuddy PG, Loftus LS. Influence of mitotane on the hypoprothrombinemic effect of warfarin. South Med J. 1986;79(3):387-8.
313. Thompson ME, Highley MS. Interaction between paclitaxel and warfarin. Ann Oncol. 2003;14(3):500.
314. Peng TR, Lee LL, Wu TW. Interactions Between Warfarin and Prednisolone: A Case Report. Am J Ther. 2017; 24(4):e494.
315. Suzuki T, Koga H, Yamazaki S, Saeki H, Tanaka H, Nishimura M, Nakaseko C, Nakasa H, Nakamura H, Ariyoshi N, Kitada M. Probable interaction between warfarin and antitumor agents used in R-ESHAP chemotherapy. Clin Ther. 2008; 30(6):1155-9.
316. Watanabe H, Itoh H, Tsuchiya Y, Miyagi N, Sugiyama T, Nakai Y, Shinozaki Y, Noguchi T, Jinbu Y, Kusama M. [Reinforcement of warfarin action in a patient administered S-1]. Gan To Kagaku Ryoho. 2015;42(1):131-3.
317. Yamamuro F, Miki A, Kondo G, Maeda T, Satoh H, Hori S, Sawada Y. Individual differences in prothrombin time-international normalized ratio variation following coadministration of the anticancer agents S-1 and warfarin: 3 case reports. Int J Clin Pharmacol Ther. 2011;49(11):700-4.
318. Marutaka M, Kubota Y, Yoshida R, Orita Y. [Difficult management of warfarin anticoagulant therapy due to S-1 administration for gastric cancer--report of a case]. Gan To Kagaku Ryoho. 2009;36(9):1561-3.
319. Moretti LV, Montalvo RO. Elevated International Normalized Ratio associated with concurrent use of sorafenib and warfarin. Am J Health Syst Pharm. 2009;66(23):2123-5.
320. Givens CB, Bullock LN, Franks AS. Safety of concomitant tamoxifen and warfarin. Ann Pharmacother. 2009;43(11):1867-71.
321. Mishra D, Paudel R, Kishore PV, Palaian S, Bista D, Misra P. Interaction between warfarin and tamoxifen: a case report. Kathmandu Univ Med J (KUMJ). 2007;5(1):105-7.
322. Tenni P, Lalich DL, Byrne MJ. Life threatening interaction between tamoxifen and warfarin. BMJ. 1989;298(6666):93.
323. Lodwick R, McConkey B, Brown AM. Life threatening interaction between tamoxifen and warfarin. Br Med J (Clin Res Ed). 1987;295(6606):1141.
324. Nissenblatt MJ, Karp GI. Bleeding risk with trastuzumab (Herceptin) treatment. JAMA. 1999;282(24):2299-301.
325. Lim S, Houranieh J, Crawford R. Elevated International Normalized Ratio in a patient concurrently using warfarin and vismodegib. Am J Health Syst Pharm. 2014;71(3):200-3.
326. Gundabolu K. Anticoagulants Could Be a Victim of Enzalutamide. J Oncol Pract. 2017 Nov;13(11):730-731. doi: 10.1200/JOP.2017.027615.
327. Le Guern A, Lebas D, Thillard EM, Martincic C, Wiart T, Modiano P. [Hemorrhagic syndrome during cobimetinib therapy: The need for caution in patients receiving new anticoagulant drugs]. Ann Dermatol Venereol. 2017; 144(5):383-384.

328. Salemis NS. Rivaroxaban-induced chest wall spontaneous expanding hematoma. Drug Discov Ther. 2017;11(1):47-50. DOI: 10.5582/ddt.2016.01064Case
329. Nassif T, Banchs J, Yusuf SW, Mouhayar E. Acute haemorrhagic tamponade in cancer patients receiving direct oral anticoagulant: case series. Eur Heart J Case Rep. 2017;1(2):ytx018.
330. Munoz J, Hong D, Kurzrock R. Anticoagulation-induced severe bleeding in a patient receiving bevacizumab therapy. Int J Hematol. 2012;95(1):1-2.

Supplementary Table 2. Adjusted logistic models of mechanisms of DDI and tyrosine kinase inhibitors

**Variáveis**	**Adjusted model**	
	**OR (95% CI)**	***P*-value**
CYP3A4 substrate	5.20 (1.29–20.89)	0.020
P-glycoprotein substrate	1.93 (0.74–4.89)	0.177
CYP3A4 competition	0.39 (0.07–2.10)	0.275
P-glycoprotein competition	–	>0.99
CYP3A4 inhibition	1.62 (0.81–3.23)	0.172
P-glycoprotein inhibition	3.99 (2.16–7.39)	<0.001
CYP3A4 induction	1.84 (0.88–3.87)	0.107
P-glycoprotein induction	–	0.999

OR: odds ratio. 95% CI: 95% confidence interval.
